# Extreme Morphology, Functional Trade-offs, and Evolutionary Dynamics in a Clade of Open-Ocean Fishes (Perciformes: Bramidae)

**DOI:** 10.1093/iob/obab003

**Published:** 2021-02-16

**Authors:** Michelle C Gilbert, Andrew J Conith, Catherine S Lerose, Joshua K Moyer, Steve H Huskey, R Craig Albertson

**Affiliations:** 1 Organismic and Evolutionary Biology Graduate Program, University of Massachusetts, Amherst, MA 01003, USA; 2 Biology Department, Morrill Science Center, University of Massachusetts, 611 North Pleasant Street, Amherst, MA 01003, USA; 3 Biology Department, Western Kentucky University, 1906 College Heights Boulevard, Bowling Green, KY 42101, USA

## Abstract

When novel or extreme morphologies arise, they are oft met with the burden of functional trade-offs in other aspects of anatomy, which may limit phenotypic diversification and make particular adaptive peaks inaccessible. Bramids (Perciformes: Bramidae) comprise a small family of 20 extant species of fishes, which are distributed throughout pelagic waters worldwide. Within the Bramidae, the fanfishes (*Pteraclis* and *Pterycombus*) differ morphologically from the generally stout, laterally compressed species that typify the family. Instead, *Pteraclis* and *Pterycombus* exhibit extreme anterior positioning of the dorsal fin onto the craniofacial skeleton. Consequently, they possess fin and skull anatomies that are radically different from other bramid species. Here, we investigate the anatomy, development, and evolution of the Bramidae to test the hypothesis that morphological innovations come at functional (proximate) and evolutionary (ultimate) costs. Addressing proximate effects, we find that the development of an exaggerated dorsal fin is associated with neurocrania modified to accommodate an anterior expansion of the dorsal fin. This occurs via reduced development of the supraoccipital crest (SOC), providing a broad surface area on the skull for insertion of the dorsal fin musculature. While these anatomical shifts are presumably associated with enhanced maneuverability in fanfishes, they are also predicted to result in compromised suction feeding, possibly limiting the mechanisms of feeding in this group. Phylogenetic analyses suggest craniofacial and fin morphologies of fanfishes evolved rapidly and are evolutionarily correlated across bramids. Furthermore, fanfishes exhibit a similar rate of lineage diversification as the rest of the Bramidae, lending little support for the prediction that exaggerated medial fins are associated with phylogenetic constraint. Our phylogeny places fanfishes at the base of the Bramidae and suggests that nonfanfish bramids have reduced medial fins and re-evolved SOCs. These observations suggest that the evolution of novel fin morphologies in basal species has led to the phylogenetic coupling of head and fin shape, possibly predisposing the entire family to a limited range of feeding. Thus, the evolution of extreme morphologies may have carryover effects, even after the morphology is lost, limiting ecological diversification of lineages.

## 解題

新規または極端な形態が発生すると、解剖学的構造の他の側面で機能的なトレードオフの負担に直面することが多く、表現型の多様化が制限され、特定の適応ピークにアクセスできなくなる可能性があります。ブラミド（スズキ目：シマガツオ科）は、20種の現存する魚の小さな家族で構成されており、世界中の遠洋水域に分布しています。ブラミダエ内では、シマガツオ（*Pteraclis*と*Pterycombus*）は、家族を代表する一般的に頑丈な横方向に圧縮された種と形態学的に異なります。代わりに、*Pteraclis*と*Pterycombus*は、頭蓋顔面骨格への背びれの極端な前方位置を示します。その結果、それらは他のブラミド種とは根本的に異なるひれと頭蓋骨の解剖学的構造を持っています。ここでは、形態学的革新が機能的（近接）および進化的（究極）コストでもたらされるという仮説をテストするために、ブラミダエの解剖学、発達、および進化を調査します。近接効果に対処すると、誇張された背びれの発達は、背びれの前方拡張に対応するように修正された脳頭蓋に関連していることがわかります。これは、後頭上頂（SOC）の発達の低下を介して発生し、背びれの筋肉組織を挿入するための頭蓋骨の広い表面積を提供します。これらの解剖学的変化はおそらくファンフィッシュの操作性の向上に関連していると思われますが、吸引餌の低下をもたらし、このグループの餌のメカニズムを制限する可能性もあると予測されています。系統発生分析は、シマガツオの頭蓋顔面およびヒレの形態が急速に進化し、ブラミド間で進化的に相関していることを示唆しています。さらに、ファンフィッシュは他のブラミダエと同様の系統多様化率を示し、誇張された内側のひれが系統発生の制約に関連しているという予測をほとんど支持していません。私たちの系統発生は、シマガツオをシマガツオ科の根元に配置し、シマガツオ以外のシマガツオが内側のひれを減らし、SOCを再進化させたことを示唆しています。これらの観察結果は、基底種における新しいヒレの形態の進化が、頭とヒレの形状の系統発生的結合をもたらし、おそらく家族全員が限られた範囲の摂食にかかりやすくなっていることを示唆しています。したがって、極端な形態の進化は、形態が失われた後でも持ち越し効果をもたらす可能性があり、系統の生態学的多様化を制限します。

## Portuguese

Quando surgem morfologias novas ou extremas, muitas vezes enfrentam o fardo de compensações funcionais em outros aspectos da anatomia, que podem limitar a diversificação fenotípica e tornar determinados picos adaptativos inacessíveis. Bramids (Perciformes: Bramidae) compreendem uma pequena família de 20 espécies existentes de peixes, que estão distribuídos em águas pelágicas em todo o mundo. Dentro dos Bramidae, os fanfishes (*Pteraclis* e *Pterycombus*) diferem morfologicamente das espécies geralmente robustas e comprimidas lateralmente que tipificam a família. Em vez disso, *Pteraclis* e *Pterycombus* exibem posicionamento anterior extremo da nadadeira dorsal no esqueleto craniofacial. Conseqüentemente, eles possuem anatomias de barbatana e crânio que são radicalmente diferentes de outras espécies de bramida. Aqui, investigamos a anatomia, o desenvolvimento e a evolução dos Bramidae para testar a hipótese de que as inovações morfológicas têm custos funcionais (proximais) e evolutivos (finais). Abordando os efeitos imediatos, descobrimos que o desenvolvimento de uma nadadeira dorsal exagerada está associado a neurocrania modificada para acomodar uma expansão anterior da nadadeira dorsal. Isso ocorre por meio do desenvolvimento reduzido da crista supraoccipital (SOC), proporcionando uma ampla área de superfície no crânio para a inserção da musculatura da nadadeira dorsal. Embora essas mudanças anatômicas estejam presumivelmente associadas a maior capacidade de manobra em peixes-leque, também se prevê que resultem em alimentação de sucção comprometida, possivelmente limitando os mecanismos de alimentação neste grupo. As análises filogenéticas sugerem que as morfologias craniofaciais e das nadadeiras de fanfishes evoluíram rapidamente e estão evolutivamente correlacionadas entre as bramidas. Além disso, fanfishes exibem uma taxa semelhante de diversificação de linhagem como o resto dos Bramidae, emprestando pouco suporte para a previsão de que nadadeiras mediais exageradas estão associadas a restrições filogenéticas. Nossa filogenia coloca fanfishes na base dos Bramidae e sugere que bramids não fanfish possuem nadadeiras mediais reduzidas e SOCs re-evoluídos. Essas observações sugerem que a evolução de novas morfologias de nadadeiras em espécies basais levou ao acoplamento filogenético da forma da cabeça e da nadadeira, possivelmente predispondo toda a família a uma faixa limitada de alimentação. Assim, a evolução de morfologias extremas pode ter efeitos de transporte, mesmo após a perda da morfologia, limitando a diversificação ecológica das linhagens.

## Spanish

Cuando surgen morfologías nuevas o extremas, a menudo se encuentran con la carga de compensaciones funcionales en otros aspectos de la anatomía, lo que puede limitar la diversificación fenotípica y hacer inaccesibles los picos adaptativos particulares. Las bramidas (Perciformes: Bramidae) comprenden una pequeña familia de 20 especies de peces existentes, que se distribuyen en las aguas pelágicas de todo el mundo. Dentro de los Bramidae, los fanfishes (*Pteraclis* y *Pterycombus*) difieren morfológicamente de las especies generalmente robustas y comprimidas lateralmente que caracterizan a la familia. En cambio, *Pteraclis* y *Pterycombus* exhiben una posición anterior extrema de la aleta dorsal sobre el esqueleto craneofacial. En consecuencia, poseen anatomías de aletas y cráneo que son radicalmente diferentes de otras especies de bramidas. Aquí, investigamos la anatomía, el desarrollo y la evolución de Bramidae para probar la hipótesis de que las innovaciones morfológicas tienen un costo funcional (próximo) y evolutivo (último). Al abordar los efectos inmediatos, encontramos que el desarrollo de una aleta dorsal exagerada se asocia con neurocráneo modificado para adaptarse a una expansión anterior de la aleta dorsal. Esto ocurre a través del desarrollo reducido de la cresta supraoccipital (SOC), proporcionando una amplia área de superficie en el cráneo para la inserción de la musculatura de la aleta dorsal. Si bien estos cambios anatómicos presumiblemente están asociados con una mayor maniobrabilidad en los peces fanfishes, también se predice que darán como resultado una alimentación por succión comprometida, lo que posiblemente limite los mecanismos de alimentación en este grupo. Los análisis filogenéticos sugieren que las morfologías craneofaciales y de aletas de los fanfishes evolucionaron rápidamente y están correlacionadas evolutivamente entre las bramidas. Además, los fanfishes exhiben una tasa similar de diversificación de linajes que el resto de los Bramidae, lo que brinda poco apoyo a la predicción de que las aletas mediales exageradas están asociadas con restricciones filogenéticas. Nuestra filogenia coloca a los peces abanico en la base de las Bramidae y sugiere que las bramidas que no son peces abanico tienen aletas mediales reducidas y SOC reevolucionado. Estas observaciones sugieren que la evolución de nuevas morfologías de aletas en especies basales ha llevado al acoplamiento filogenético de la forma de la cabeza y la aleta, lo que posiblemente predisponga a toda la familia a un rango limitado de alimentación. Por lo tanto, la evolución de morfologías extremas puede tener efectos de arrastre, incluso después de que se pierde la morfología, lo que limita la diversificación ecológica de los linajes.

## Introduction

When a novel trait is manifested, it not only must work in the confines of previous constraints (historical contingency), but it also introduces new constraints to the system (Jacob 1977), which can limit evolutionary trajectories by restricting the number of adaptive peaks that can be reached by a lineage (Wright 1932; Arnold 1992; Schluter 1996). Darwin (1859) acknowledged the impact evolutionary histories and developmental processes have on evolutionary trajectories, noting that the interplay of the two results in a “unity of type.” Given that organisms use morphological structures to complete numerous ecologically relevant tasks (e.g., feeding, locomotion, reproduction), and no single phenotype enables optimal performance in all tasks, a structural dilemma exists, forcing evolutionary trajectories to optimize phenotypes via compromise (Arnold 1983). Pareto optimality theory, historically used in the fields of engineering and economics, suggests that a multidimensional phenotype cannot be improved for all tasks at once (McGhee 2007; Kennedy 2010; Shoval et al. 2012) and has been increasingly used in biology to explain evolutionary constraints that limit phenotypic evolution (Farnsworth and Niklas 1995; Sheftel et al. 2013; Tendler et al. 2015). To understand the current and future evolutionary and phenotypic trajectories of a species, one must consider the trade-offs that have deflected past trajectories to produce the observed phenotype.

Phenotypic trade-offs have been a core component of evolutionary biology for decades (Charnov 1989; Stearns 1989; Leroi et al. 1994; Brodie and Brodie 1999; Patek and Oakley 2003; Roff and Fairbairn 2007). Specialization and the consequential performance/functional trade-off(s) have been documented across numerous taxa (Toro et al. 2004; Langerhans et al. 2005; Herrel et al. 2009; Herrel and Bonneaud 2012; Holzman et al. 2012; Pelegrin et al. 2017), and, at times, can appear inconspicuous. However, when morphological traits are exaggerated, the demand on the system as a whole is greater, forcing trade-offs to be more substantial. Such is the case for *Tropidurus* lizards in Northeastern Brazil, where specialized rock-dwelling ecomorphs are dorsoventrally flattened to aid in traversing narrow rocky crevasses, but suffer from a 66% loss in overall egg capacity (Pelegrin et al. 2017). In the carabid beetle, *Damaster blaptoides*, two diametrically distinct head morphologies are observed depending on the shell size of resident snails. Konuma and Chiba (2007) report that beetle populations with small heads are able to consume snail prey directly by reaching into the aperture, but this forces the size of mandibles and associated muscles to be significantly reduced. In bony fishes, one would expect extreme jaw protrusion to lead to greater suction feeding capabilities. However, the mechanism of extreme premaxillary protrusion in two cichlid species significantly decreases suction feeding performance and, instead, appears to be an adaptation that optimizes ram feeding on elusive prey (Waltzek and Wainwright 2003). While there are numerous studies that aim to address proximate (e.g., functional, biomechanical) or ultimate (e.g., evolutionary constraints) consequences of such trade-offs, few are able to connect the two due to the difficulty in resolving long-term evolutionary history with contemporary functional studies. Here, we seek to test the hypothesis that extreme morphological traits result in, not only functional trade-offs, but also long-term evolutionary trade-offs (as constraints). To investigate this, we explore the development, anatomy, and phylogenetic relationships of a unique clade of fishes in the family Bramidae, the fanfishes.

Bramids (Perciformes: Bramidae) are a small family of fishes comprised of 20 extant species across seven genera. Nearly all bramids are known, or thought, to be migratory, traversing the high seas seasonally for food and reproduction (Mead 1972). Despite this, and having representatives in every major ocean (Mead 1972), they remain uncommon and, in some taxa, quite rare. Much of the contemporary work concerning bramids is isolated to sightings and bycatches that provide new information on their distribution (Gutiérrez et al. 2005; Park et al. 2007; Carvalho-filho et al. 2009; Ali and McNoon 2009; González-lorenzo et al. 2013; Jawad et al. 2014; Lee and Kim 2015; Orr et al. 2018; Lee et al. 2019; Rahangdale et al. 2019), insights to their ecology (Lobo and Erzini 2001; Moteki et al. 2001; Carvalho-filho et al. 2009), and opportunities to obtain mitochondrial sequence data (Chen et al. 2016; Liu et al. 2016; Xu et al. 2018). Within the family, two sister genera, *Pterycombus* and *Pteraclis* (commonly known as fanfishes), stand as outliers, deviating from the generally stout, laterally compressed morphologies that typify the family. Instead, these two genera are characterized by relatively elongate bodies and extreme anterior extensions of dorsal and anal fins, extending well onto the neurocranium and even beyond the orbit in some species. Work detailing the anatomy and evolutionary interrelationships of the family are scarce or limited to a select few taxa and it is unknown how the exaggeration of the dorsal fin has influenced, if at all, the neurocranium. Mead echoed this in his 1972 monograph, stating “The phyletic unity of these six remains in doubt and this question, together with that of the origin of the group, deserves further study,” referring to the six genera that were known at the time, as *Xenobrama* went undescribed until 1989 (Yatsu and Nakamura 1989).

The goal of this study was to identify possible functional and/or biomechanical trade-offs associated with extreme, morphological adaptations, determine whether there are regions of bramid morphology that have been constrained through carry over effects from their evolutionary history, and assess how early during ontogeny these differences are detectable. The unique anatomy of the fanfishes offers an opportunity to investigate how extreme morphologies can not only impose proximate trade-offs in functional morphology, but also constrain evolution and levy evolutionary trade-offs. To this end, we wanted to understand whether the extreme dorsal fin morphology in fanfishes has influenced the evolutionary trajectory of the family by introducing phenotypic constraints that deflected historical trajectories or limited diversification.

## Methods

### Phylogenetic tree construction

We utilized the mitochondrial genes for cytochrome oxidase subunit 1 (COI) and Cytochrome B (cytb) retrieved from GenBank (Benson et al. 2013) in keeping with the methods of recent studies of mitochondrial sequence data across the family Bramidae (Chen et al. 2016; Liu et al. 2016; Xu et al. 2018). Accession numbers for all gene data are provided in Supplemental Table 1. Both COI (length ∼640 bp) and cytb (length ∼1141 bp) were aligned using the AliView v1.25 alignment viewer and editor (Larsson 2014). We constructed a Bayesian, time-calibrated tree of all available bramid taxa listed on GenBank, encompassing 14/20 known species (*n* = 43), including a representative of the closely related family Caristiidae (*Caristius macropus*, *n* = 1), and three representative species from the family Stromateidae (*Peprilus paru*, *n* = 1; *Peprilus simillimus*, *n* = 3; *Peprilus triacanthus*, *n* = 6) as an outgroup.

To conduct a Bayesian, time-calibrated analysis of the Bramidae, we constructed an XML input file for BEAST using the BEAUTi v.2.5.1 application (Bouckaert et al. 2014). We used the bModelTest v.1.1.2 application to estimate substitution models for these mitochondrial genes. We selected the default transition–transversion split option, which allows BEAST to average out uncertainty in substitution model selection during the Markov chain Monte Carlo (MCMC) run (Bouckaert and Drummond 2017). Based on AICc fit, bModelTest selected different substitution models for each codon partition. Codon positions for each gene, following gene alignment, are as follows: COI position 1: 121131; COI position 2: 121321; COI position 3: 121134; cytb position 1: 123421; cytb position 2: 123343; cytb position 3: 121123.

We used a log-normal distributed relaxed molecular clock for divergence time estimation and assigned a pure-birth (Yule) model as the branching process. All other parameters we left at their default settings. To estimate divergence times, we used a series of fossil calibrations outlined by Miya et al. (2013). We set the split between Caristiidae and Bramidae (log normal distribution; offset = 56.0, mean = 1, lower = 0.0, upper = 0.72) at 56 mya (Bannikov and Tyler 1994; Fierstine et al. 2012), crown Bramidae (log normal distribution; offset = 49.11, mean = 1, lower = 0.0, upper = 2.0) at 49.11 mya (Casier 1966; Ellison et al. 1994; Baciu and Bannikov 2003), and crown Stromateidae (log normal distribution; offset = 31.35, mean = 1, lower = 0.0, upper = 5.0) at 31.35 mya (Lenov 1998; Bannikov 2012). Finally, we performed four independent runs for 2 × 10^7^ generations sampling every 1000 generations using the BEAST v.2.5.1 module (Bouckaert et al. 2014) on the CIPRES Science Gateway v3.3 computing cluster (Miller et al. 2010).

We used Tracer v1.7.1 (Rambaut et al. 2018) to test for convergence of our four runs, and used effective sample size to check the true posterior and likelihood distributions. We removed 20% for burn in using Log Combiner v2.5.1 and the maximum clade credibility tree (MCCT) was created using TreeAnnotator v2.5.1 (Drummond et al. 2006).

### Morphometric data collection

Given the difficulty of acquiring bramid specimens, and the rarity of the fanfishes in general, we utilized the collection of the Museum of Comparative Zoology at Harvard University (Cambridge, MA, USA) to obtain representative specimens of the family Bramidae. A single *Eumegistus illustris* was obtained from the Smithsonian National Museum of Natural History (Washington DC, USA), and two specimens of *Pteraclis aesticola* were obtained from the Australian Museum of Natural History (Darlinghurst, Australia). A single intact *Pterycombus petersii* specimen was collected when it was regurgitated by a yellowfin tuna (*Thunnus albacares*) off the coast of Hawaii (see Acknowledgments section). The combination of the previous factors limits one’s ability to conduct proper kinematic studies. Therefore, we focus on the aspects of functional morphology in our questions and interpretations. Details on all lots, adult and juvenile, can be found in Supplemental Table S2. As they were not the focus of this study, we did not collect morphometric data concerning Stromateidae.

We photographed the left-lateral surface of museum specimens, with the exception of two adult *Pteraclis aesticola* and a single *Eumegistus illustris*, for which photographs were obtained through web portals. All available adults and a number of juveniles representing the available genera were utilized (Supplemental Table S2).

**Fig. 1:**
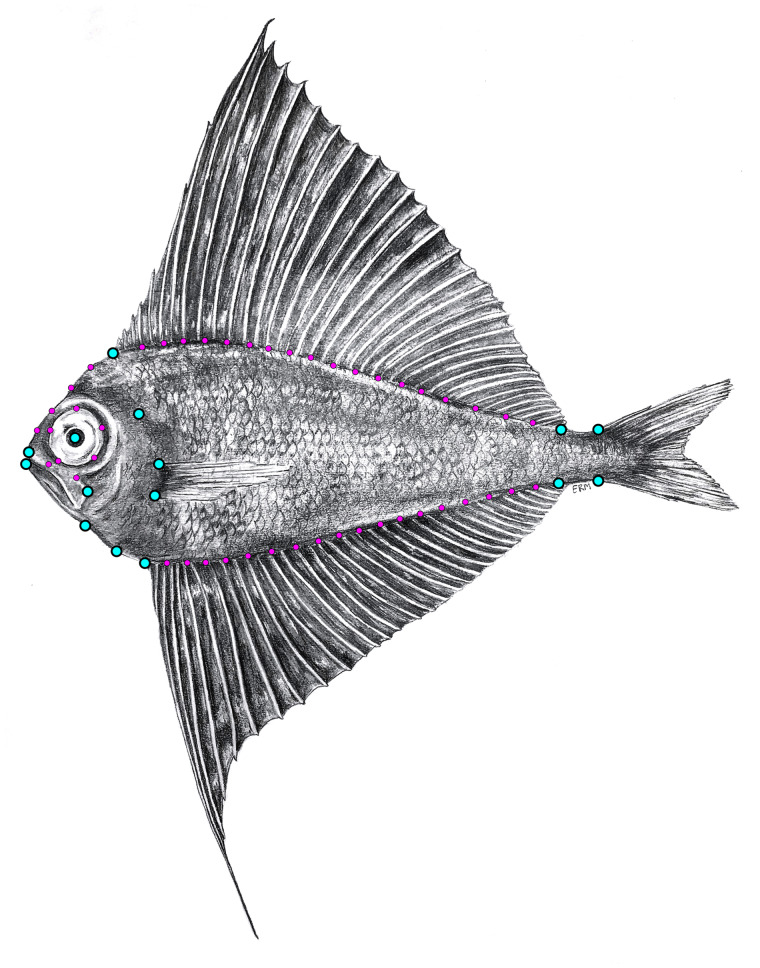
Illustration of a fresh *Pterycombus petersii* specimen with the landmark configuration used in this study. In total, we used 62 landmarks, 15 of which were fixed (cyan), the remaining 47 being semi-(sliding)-landmarks (magenta). Fixed landmarks are placed on the: tip of premaxilla, dorsal fin insertions (nape being listed here as the region between tip of premaxilla and dorsal fin insertion), dorsal- and ventral-most point where the caudal peduncle meets the caudal fin, anal fin insertions, pelvic fin insertion, anterior margin of breast (breast is defined as the margin of opercle to pelvic fin insertion), dorsal and ventral pectoral fin insertion, dorsal end of the opercular opening, anterior tip of the dentary, posterior tip of maxilla, and center of the eye. Illustration hand drawn by Emma R. Masse

Our morphological landmark configuration (Fig. 1) consisted of 15 fixed anatomical landmarks and 47 sliding semi-landmarks and was subjected to generalized Procrustes analysis (Goodall 1991) utilizing bending energy. These data were later parsed into two separate configurations: head and body shape. Our configuration for body shape is largely driven by body depth, length, and fin length, with dorsal and anal fin base length being the primary trait of interest. Head shape configuration was largely driven by nape size, maxilla length and angle, and eye placement. All coordinate data were collected via STEREOMORPH (Olsen and Westneat 2015) in R (R Core Team 2018).

Additionally, we collected linear measures of all genera for which we had three or more specimens, focusing specifically on lower jaw (the tip of the dentary to the mandible quadrate joint) and head length (tip of snout to the furthest posterior margin of the operculum). These data were collected from digital photographs using the software package MorphoJ (Klingenberg 2011). We then regressed lower jaw length against head length to look for differences across the Bramidae. These data were log transformed and plotted twice, once to evaluate the family as a whole and once to calculate regression lines for each of the four genera.

### Phylogenetic comparative methods

The MCCT was pruned of all bramid taxa for which we did not have both morphometric and phylogenetic information (*n* = 2, *Xenobrama microlepis* and *Brama australis*), leaving 12 bramid taxa and a single representative of Caristiidae, *Caristius sp*. Since we did not have phylogenetic data for *C. macropus*, we matched those data with morphological data from the only congener we could acquire, *C. fasciatus.* This pruned topology provided the framework for all subsequent comparative analyses.

Using GEOMORPH v3.0.6 (Adams et al. 2014, 2018) and PHYTOOLS (Revell 2012), the consensus tree and morphometric data were used to generate a phylomorphospace that mapped principal component (PC) data for morphology with the phylogenetic relationships intertwined. We used the combination of two.b.pls and phylo.integration (Rohlf and Corti 2000; Adams and Felice 2014; Collyer et al. 2015; Adams and Collyer 2016, 2018) functions in GEOMORPH to assess the association between the head and body configurations. The functions use partial least squares to estimate the degree of covariation between our two variables, while the latter does so while also accounting for the phylogeny under a Brownian motion model of evolution. We used two approaches to characterize the Brownian rate of morphological evolution in the Bramidae. First, we used the compare.evol.rates function in GEOMORPH to assess the rate of morphological evolution between the bramids and fanfish clades. Second, we used the compare.multi.evol.rates function in GEOMORPH to assess rates of morphological evolution in the head and body configurations independently (Denton and Adams 2015). Both these methods estimate phylogenetically corrected rates based on a distance approach for high-dimensional datasets such as shape (Adams 2014).

We calculated the mean PC1 and PC2 scores for head and body shape across each taxon and then determined rates of trait evolution across the phylogeny. To accomplish this, we utilized routines contained within the Bayesian analysis of macroevolutionary mixtures (BAMM) software package (Rabosky et al. 2013; Rabosky 2014). BAMM analysis was executed with four reversible jump MCMC simulations for 1 × 10^7^ generations, sampling every 1000 generations. Our prior distributions were estimated via BAMMtools (Rabosky et al. 2014) in R (R Core Team 2018). This was repeated for PC1 and PC2 means for head (βIntPrior: 317.220 & 2103.425; βShiftPrior: 0.023 & 0.023) and body (βIntPrior: 510.455 & 2410.561; βShiftPrior: 0.023 & 0.023) shape for the entire phylogeny. BAMM output files were then also analyzed with BAMMtools (Rabosky et al. 2014).

To investigate further the diversification dynamics of the family Bramidae, we used the gamma (γ) summary statistic to characterize lineage diversification through time (Pybus and Harvey 2000) using the full phylogeny. Given incomplete taxon sampling (six missing taxa; *Brama caribbea*, *B. myersi*, *B. pauciradiata*, *Eumegistus brevorti*, *Pteraclis carolinus*, and *P. velifera*), we assessed γ using the Monte Carlo constant-rates (MCCRs) test. This test uses our bramid data to simulate 5000 phylogenies under a constant-rate pure-birth diversification model (the null), before randomly pruning taxa from the simulated trees to mimic incomplete sampling and derive a null distribution of γ statistics. The MCCR test then compares the empirical γ value to the simulated distribution to generate a *P* value.

We then compared the fit of four different diversification models to our tree. We assessed two rate constant models, pure-birth (Yule) and constant-rate birth-death, and two rate-variable models, variable-rate density-dependent logistic (DDL) and a variable-rate exponential density-dependent (DDX) model of lineage diversification (Rabosky and Lovette 2008). These analyses use Birth-Death likelihoods, which offer an advantage over the γ statistic alone when background extinction rates are nonzero (Rabosky 2006). Models were statistically evaluated with the Akaike information criterion (AIC). To visually reflect these patterns, we constructed a lineage-through-time (LTT) plot from the MCCT.

We then explicitly tested for differences in the rate of lineage diversification between fanfish and the remaining bramids. We scored the presence or absence of elongated fin morphology as binary characters and estimated state-specific speciation and extinction rates in a Bayesian framework (Fitzjohn 2012). Specifically, we assessed the diversification rate in each group using the binary state speciation and extinction (BiSSE) model from the R package diversitree (v.0.9-14). We set exponential priors for each parameter in BiSSE with rate 1/(2*r*), where *r* is the trait-independent diversification rate. Maximum-likelihood-estimated model parameters served as a starting point. MCMC chains were run for 5000 generations, and we discarded the initial 10% as burn-in. To account for incomplete taxon sampling, we used the sampling fraction procedure, which requires the specification of the number of taxa present in each grouping out of the total number of described species in that group (elongated fins absent = 0.73, elongated fins present = 0.6).

### Bramid ontogeny

To determine ontogenetic differences in shape, we used geometric morphometrics to quantify and determine phenotypic trajectories (Collyer and Adams 2013; Collyer et al. 2015). Ontogenetic trajectories can provide valuable insights into the developmental mechanisms and processes that facilitate phenotypic evolution. Specifically, our aim was to determine how early morphological difference arose across the family Bramidae. We assessed two stages of development across four of the seven bramid genera (excluding *Eumegistus*, *Xenobrama*, and *Pteraclis* due to extreme difficulty in acquiring both juvenile and adult specimens) and a very limited sample of *Caristius fasciatus* for outgroup comparisons. We digitized individuals in both developmental stages following the same landmarking scheme as the adults (see Fig. 1). Phenotypic trajectories were evaluated via trajectory.analysis (Collyer and Adams 2013; Collyer et al. 2015) in GEOMORPH. This function evaluates phenotypic trajectories through the use of analysis of variance (ANOVA) and a Randomized Residual Permutation Procedure (Collyer and Adams 2018), calculating differences in trajectory path and magnitude. In our model (Shape ∼ Genus * Stage, ∼ Centroid Size), we included size as a covariate and deemed it to be an outside source of shape variation. These outside sources of variation are accounted for prior to the trajectory defining variables of genera and developmental stage. Using the results from a PC analysis, we mapped ontogenetic trajectories into morphospace using the first two PCs.

In addition to geometric morphometrics and with permission from the Harvard MCZ, *Pterycombus brama* and *Brama dussumieri* larvae were cleared and stained across early and late juvenile stages to identify anatomical differences. Images were captured with both LED backlights and, to take advantage of the fluorescent properties of alizarin, under fluorescent light with a red fluorescent protein (RFP) filter. By using fluorescent lighting and an RFP filter, we were better able to isolate the ossified elements in the craniofacial skeleton and identify anatomical elements of interest.

### Craniofacial anatomy

Because ecological, functional, and behavioral data are limited, we chose to investigate the osteology and myology of the rare *Pterycombus petersii* (see Acknowledgments section) to glean insights into the functional and ecological properties of the genus and, ideally, family. To accomplish this, we used a combination of X-ray micro-computer tomography (µCT) and gross anatomization. We used a Bruker Skyscan 1276 µCT (Bruker microCT, Kontich, Belgium) at the University of Massachusetts Animal Imaging Core (Amherst, MA) to collect high-resolution scans of *P. petersii*. We scanned at 20-µm resolution with a 0.25-mm aluminum filter. Reconstruction was accomplished with the use of InstaRecon CBR (Bruker, Kontich, Belgium). Z-stack images were oriented and cropped with IrfanView v4.54 (Irfan Skiljan, Austria), skeletal anatomy was segmented using Mimics v19 (Materialise NV, Leuven, Belgium). We then exported mesh models to Geomagic 2014 v1.0 (3 D Systems, Rock Hill, SC, USA) to remove noise and ultimately visualized using MeshLab 2016 (Cignoni et al. 2008).

To better visualize osteological elements and associated muscles, we double stained an intact *Pterycombus petersii* specimen in alcian and alizarin. The alizarin stain was dissolved in a 75% ethanol solution, rather than the typical 0.25%–1% potassium hydroxide to preserve muscle integrity, color, and form, specifically to visualize epaxial and dorsal fin musculature attachment points on the neurocranium. Enough alizarin was added to the ethanol solution to turn it a modest orange color. The specimen was stained overnight and rinsed in 95% ethanol the following morning until the solution remained clear. Pigment bleaching and clearing phases were skipped altogether, and the specimen was stored in 75% ethanol. We then performed careful dissections across the specimen to identify skeletal elements and muscles of interest, especially those involved in dorsal fin adduction and abduction and muscles associated with feeding (e.g., adductor mandibulae, dilatator opercula, levator arcus palatini [Gosline 1971; Liem and Osse 1975; Westneat 2004; Datovo and Vari 2013]). Images were recorded using a Leica M165 FC microscope and attached Leica DFC450 camera (Leica Camera AG, Wetzlar, Germany). Post-processing (manipulation of contrast, brightness, and focus image stacking) of all images was conducted in Adobe Photoshop CC 2019 (Adobe Systems, San Jose, CA, USA).

## Results

### Bramid phylogeny

**Fig. 2:**
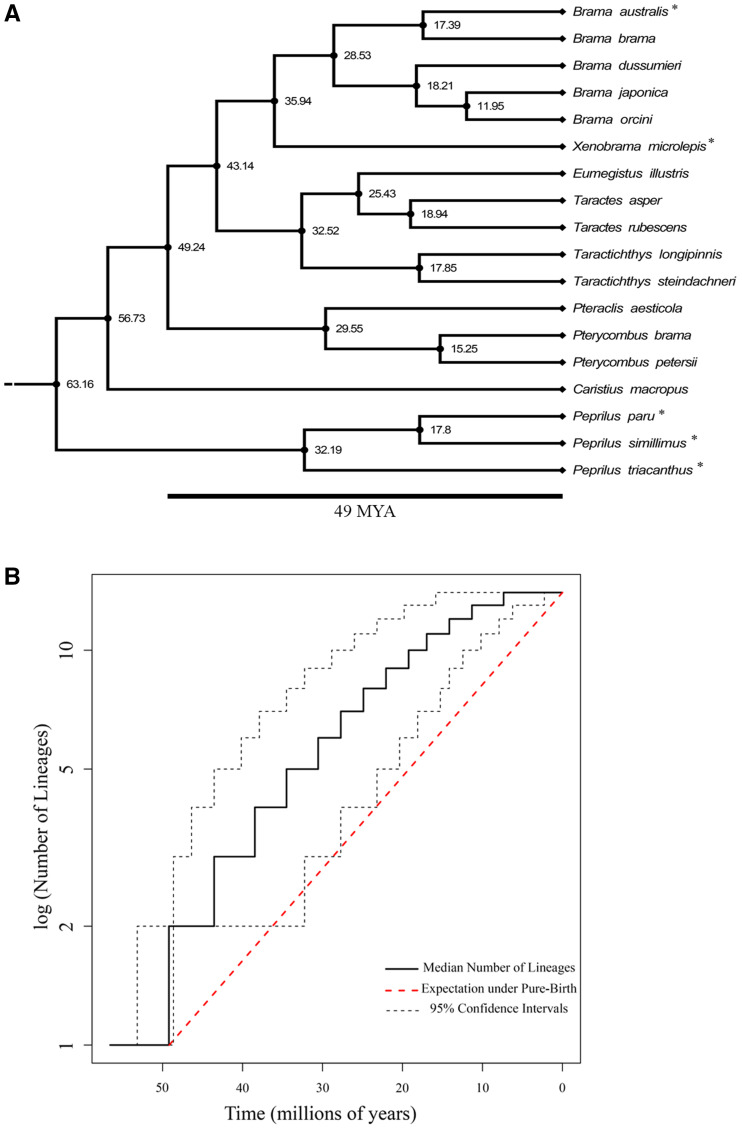
(**a**) Trimmed tree with all bramids with genetic data, and including a representative of Caristiidae. Butterfishes (Stromateidae) were used as a reliable outgroup. Asterisks indicate species that were removed from all morphometric analyses due to either genetic or morphological data being unavailable. Values indicate divergence times (mya). (**B**) Log LTT plot for the Bramidae only, excluding the stromateids and caristiids. Solid black line indicates median lineage though time curve for the consensus tree, and black*-*dashed lines illustrate 95% confidence intervals of lineages through time derived from a posterior distribution of 1000 phylogenetic trees. Red*-*dashed line depicts rate of lineage accumulation expected under constant-rate pure-birth diversification (i.e., no extinction). Bramid lineages accumulate quickly relative to a pure-birth model, before hitting a plateau as diversification slows

To assess the extent to which extreme morphologies have imposed evolutionary constraints in bramids, we first sought to reconstruct the ancestral state in the family. We find that the relationships and divergence times of the bramids included in this study (Fig. 2A; Supplemental Figure S1) are congruent with previously published trees (Miya et al. 2013; Chen et al. 2016; Friedman et al. 2019). Nodes are generally well-supported with high posterior probabilities (%PP), especially those associated with genus level relationships (%PP = >95%). Posterior support between the *Brama* & *Xenobrama* clade and *Taractes* & *Tarachtichthys* clade was lower (%PP = 77%). Support for *Eumegistus* belonging to the *Taractes* clade, as opposed to *Tarachtichthys*, was low (%PP = 49%), but support for a *Taractes*, *Tarachtichthys*, *Eumegistus* clade was high (%PP >95%). *Pteraclis* and *Pterycombus* expressed high posterior support for being part of a single clade (%PP > 95%) and were revealed to be the oldest bramid lineage (%PP >95%).

Results from the MCCR test suggest diversification rates across the Bramidae have declined through time. The γ test statistic for the MCC tree (–2.38) quantitatively demonstrates significant declines in diversification rates (*P* = 0.009) and appears robust to missing taxa (γ crit. = –1.92; *P* = 0.018). The LTT plot visually illustrates a rapid increase in lineages early in the clade’s history relative to the expectation under a constant-rate pure-birth model (Fig. 2B).

We found strong support for a density-dependent pattern of diversification in bramids (Table 1). Specifically, the DDL model best-fit the pattern of bramid lineage accumulation through time. Competing diversification models were more than four ΔAICc units away. Diversification rate parameter estimates for the DDL model (λ_0_ (initial rate) = 0.13, *K* (carrying capacity) = 13.78) suggest an initial burst of diversification followed by a linear decline in speciation rate. Lastly, we found no evidence for differences in state specific diversification rates; both the fanfish and the remaining bramids exhibit similar rates of lineage diversification (Fig. 2C; Supplementary Table S3). These data demonstrate that fanfishes represent the ancestral state, with exaggerated medial fins and laterally compressed body shape morphologies.

**Table 1:** Diversification models are ranked from best to worst based on AIC weights (wtAIC).

Model	LH	AIC	ΔAIC	wtAIC
DDL	–26.56569	57.131	0	0.883
DDX	–28.93567	61.871	4.74	0.083
Pure-birth	–31.13453	64.269	7.138	0.025
Birth-death	–31.13453	66.269	9.138	0.009

Model comparison demonstrates high support for density-dependent clade growth using a logistic model (DDL). Log likelihood (LH) is also provided for each model, as is the AIC score (AIC), and change in AIC score (ΔAIC)

### Fanfishes deviate from common bramid morphospace

We next sought to more formally characterize patterns of morphological divergence across bramids. To this end, we conducted a Procrustes ANOVA to determine the effects of size and species across the available species. Both size and species had significant effects on shape (*P* < 0.0001). Species effects explained a greater proportion of the morphological variance (*R*^2^ = 0.67) than did size alone (*R*^2^ = 0.21) and much more than the interaction of size and species (Shape ∼ size * species; *R*^2^ = 0.02). Subsequent pairwise comparisons revealed significant differences between nearly all fanfish comparisons with the other bramid taxa, and such comparisons always resulted in the greatest effect sizes (*z*-scores; Table 2). *Pterycombus brama* possessed the least number of significantly different comparisons of the fanfishes (only 6 out of 12 were significant), whereas *Pteraclis aesticola* and *Pterycombus petersii* expressed significantly different shapes in 11/12 and 10/12 comparisons, respectively. Those comparisons that were not significant were between fanfishes. Additionally, *Brama japonica* and *Taractes rubescens* also exhibited 7/12 significant pairwise comparisons.

**Table 2:** Results of Procrustes MANOVA across all available bramid species, including a single representative of Caristiidae (*Caristius fasciatus*)

	*B. brama*	*B. dussumieri*	*B. japonica*	*B. orcini*	*C. fasciatus*	*E. illustris*	*P. aesticola*	*P. brama*	*P. petersii*	*T. asper*	*T. rubescens*	*T. longipinnis*	*T. steindachneri*
*B. brama*		–0.699	–1.177	–0.356	1.480	–0.106	**7.050**	1.466	**3.138**	0.740	**1.839**	0.463	0.092
*B. dussumieri*	0.7477		–0.943	–1.408	1.351	–0.747	**5.483**	1.293	**3.592**	–0.647	–0.003	0.289	–0.344
*B. japonica*	0.9786	0.8628		–0.519	**1.585**	–0.011	**8.456**	**1.749**	**3.251**	1.273	**3.952**	**2.142**	**2.477**
*B. orcini*	0.5564	0.9971	0.6673		1.387	–0.345	**6.204**	1.381	**2.968**	–0.049	1.012	1.007	0.474
*C. fasciatus*	0.0577	0.0770	**0.0492**	0.0759		0.491	**4.109**	1.324	**2.619**	1.323	1.198	1.469	1.384
*E. illustris*	0.4094	0.7919	0.3431	0.5550	0.1917		**4.800**	1.523	**2.230**	–0.715	–0.468	0.615	0.231
*P. aesticola*	**0.0001**	**0.0003**	**0.0001**	**0.0001**	**0.0121**	**0.0027**		**1.808**	0.888	**7.781**	**8.906**	**8.548**	**9.032**
*P. brama*	0.0567	0.0783	**0.0400**	0.0733	0.0685	0.0657	**0.0473**		–0.051	**2.236**	**2.966**	**1.965**	**2.186**
*P. petersii*	**0.0168**	**0.0051**	**0.0179**	**0.0211**	**0.0337**	**0.0438**	0.1421	0.3997		**3.398**	**3.567**	**3.975**	**3.601**
*T. asper*	0.1850	0.7140	0.1020	0.4139	0.0688	0.8350	**0.0001**	**0.0341**	**0.0123**		–0.122	**2.380**	1.725
*T. rubescens*	**0.0544**	0.4207	**0.0013**	0.1331	0.0793	0.6744	**0.0001**	**0.0289**	**0.0091**	0.4641		**3.930**	**5.079**
*T. longipinnis*	0.2465	0.3070	**0.0349**	0.1261	0.0566	0.1294	**0.0001**	**0.0344**	**0.0068**	**0.0329**	**0.0031**		–0.401
*T. steindachneri*	0.3596	0.5737	**0.0198**	0.2288	0.0606	0.2011	**0.0001**	**0.0326**	**0.0116**	0.0713	**0.0002**	0.5903	

MANOVA was conducted with 10,000 permutations of residual values (Randomized Residual Permutation Procedure). Effect sizes are above and *P* values are below the diagonal. Bolded *P* values and *z* scores indicate significant differences in mean shapes between species. For the purpose of significance testing, α = 0.05.

We next trimmed the MCCT to include only species for which we possessed both mitochondrial and morphological data (Fig. 2A). The first two axes of our phylomorphospace, based on this tree, explained 62.9 and 19.6% of variation in our data, respectively (Fig. 4). The first PC (primarily representing dorsal and anal fin insertion and length, and the size of the nape, or region prior to dorsal fin insertion) completely isolated the fanfishes (*Pteraclis aesticola*, *Pterycombus brama*, *Pterycombus petersii*) from all other bramids, largely attributed to their unique dorsal and anal fin morphology. On this axis, the fanfishes possessed positive scores, while the other bramids possessed largely negative scores. The second PC axis primarily explained nape curvature, relative eye size, and body depth, with positive scores representing deeper bodies, relatively smaller eyes, and more rounded napes and negative scores being associated with shallower napes and bodies, and relatively larger eyes. Fanfishes excluded, the second axis tended to isolate genus specific groups, distinguishing the deeper bodied, highly laterally compressed *Tarachtichthys* from the slender, fusiform *Taractes*, and the more closely related *Eumegistus*. *Brama* were largely intermediate along this axis, alongside *Caristius fasciatus* and the fanfishes. In short, four distinct groups are identified in morphospace: highly laterally compressed (*Taractichthys)*, highly fusiform (*Taractes, Eumegistus*), intermediate (*Brama*), and elongate + exaggerated medial fins (*Pteraclis, Pterycombus*).

**Fig. 3:**
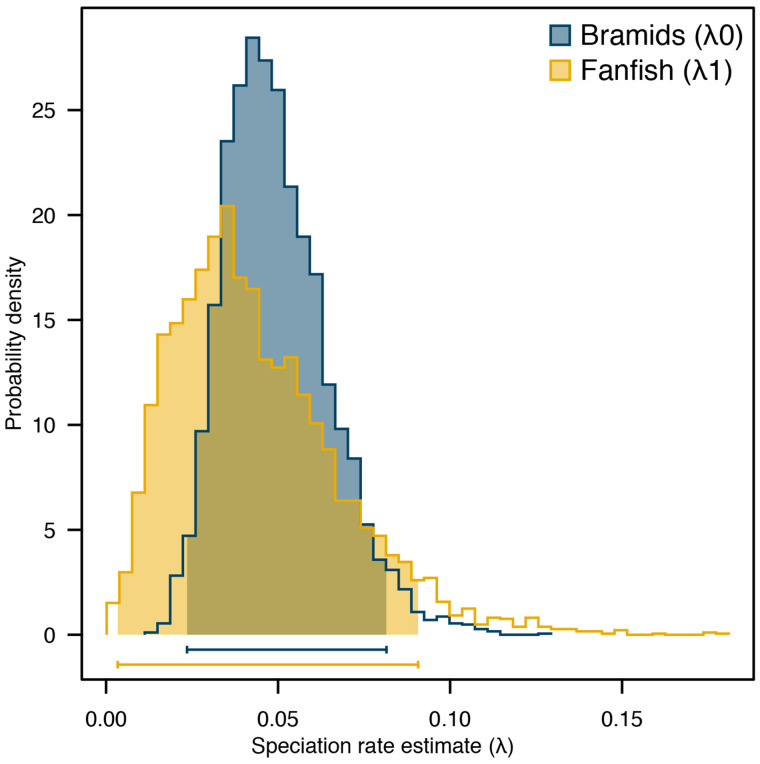
State*-*specific diversification rates between fanfishes and the remaining bramid genera. Both fanfishes and their bramid relatives exhibit substantial overlap in their speciation rate estimate distributions, suggesting similar rates of lineage diversification.

**Fig. 4:**
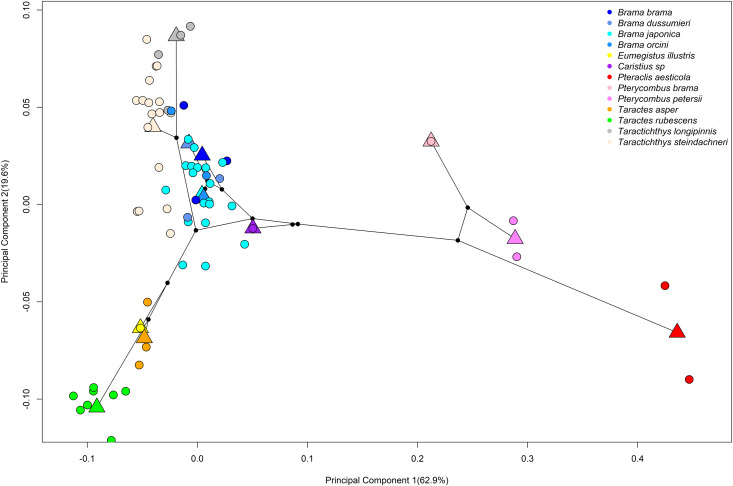
Phylomorphospace of overall body shape showing clustering of all bramid genera in negative PC space on PC1, except for the fanfishes, *Pteraclis* and *Pterycombus*, which exhibit positive PC scores on PC1. PC2 mainly separates *Taractes* (negative PC2 scores) from *Taractichthys* (positive PC2 scores). *Caristius spp* (Perciformes: Caristiidae; purple), which represents the closest related family to that of the Bramidae, exhibits a shape that is between the two major groups of bramids. Species are separated by colors, circles represent individual specimens, and triangles represent the mean shape for their respective species. Circles that exist with triangles indicate that a single specimen was available for inclusion in the analysis

These results show that fanfishes are morphologically unique when compared with the other bramids, as well as to the sister group, and that this difference is driven by their extreme exaggerated medial fin morphology.

### Head and body shapes are integrated

A possible outcome of evolutionary constraint is the integration of anatomical units, which in turn can bias the direction of morphological evolution. Since the exaggerated medial fins in fanfishes grossly extend well into the cranial region, we reasoned that this could lead to the evolutionary coupling of these two anatomical regions. Two-block partial least-squares test, without accounting for the phylogeny, revealed that head and body shape were indeed highly integrated (rPLS = 0.8445, *P* ≤0.0001; Supplemental Figure S2A). Relatively large eyes, heavily reduced nape and breast, smaller opercles, and smaller pectoral fins corresponded to a slender body, elongated dorsal and anal fins, and a small caudal fin base. Conversely, large heads and small eyes, a robust nape and breast, and large opercula corresponded to deeper bodies, relatively shorter dorsal and anal fins, and a more robust caudal fin base. This trend strengthened once we accounted for the phylogeny (rPLS = 0.9829, *P* ≤0.0001; Supplemental Figure S2B).

These results support our prediction that head and body shapes are related, and that this relationship is likely driven by expanded medial fin architecture. They are also congruent with patterns reveal by our phylomorphospace and collectively point to an evolutionary constraint that has biased the direction of morphological evolution in this group.

### Fanfishes exhibit faster rates of morphological evolution than other bramids

**Fig. 5:**
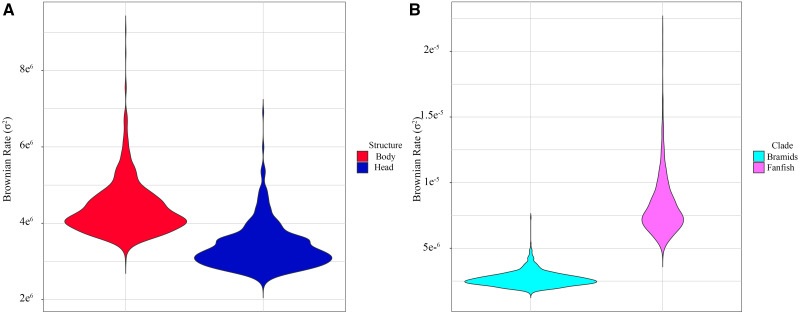
Violin plots depicting the Brownian rate of morphological evolution in the Bramidae. (**A)** Comparing rates of morphological evolution in the head and body regions of the Bramidae. (**B)** Comparing rates of morphological evolution in the fanfishes and remaining bramids

To determine whether putative constraints have also influenced rates of morphological evolution, we assessed this parameter in bramids. We found that fanfishes have experienced whole body shape evolution at a rate ∼2.93 times faster than the sum of the other bramids (rate = 8.14×10^−6^, 95% CI = 5.54×10^−6^, 1.37×10^−5^ versus rate = 2.66×10^−6^, 95% CI = 1.87×10^−6^, 4.15×10^−6^, *P* = 0.0099; Fig. 5). When head and body shapes were parsed, net rates of morphological evolution between the two units were not significantly different (head rate = 3.38 × 10^−6^, 95% CI = 2.67 × 10^−6^, 4.85 × 10^−6^, body rate = 4.38 × 10^−6^, 95% CI = 3.54 × 10^−6^, 6.10 × 10^−6^, observed rate ratio = 1.30, *P* = 1; Supplemental Figure S3).

**Fig. 6:**
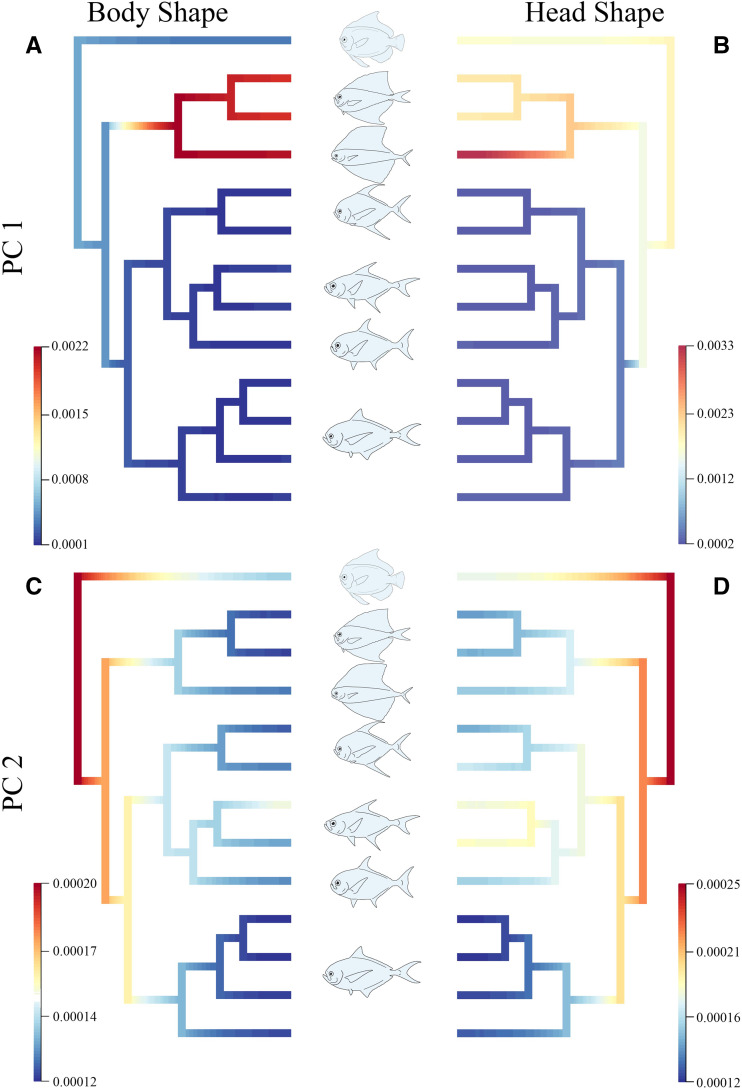
Evolutionary rates for mean body and head shape across the family Bramidae, and including a single member of Caristiidae (*Caristus sp.*). PC1 for body (A) and head (B) shape evolution. PC2 for body (C) and head (D) shape evolution. Generalized body shapes for each of the genera included in our analyses are in the center. Genera from top to bottom: *Caristus*, *Pterycombus*, *Pteraclis*, *Taractichthys*, *Taractes*, *Eumegistus*, *Brama.* Warm colors indicate faster rates of shape evolution, while cool colors represent slower rates of shape evolution

We next calculated evolutionary rates for head and body shape independently in an attempt to tease apart any taxon-specific differences with mean PC1 and PC2 scores representing the traits (Fig. 6). For body shape, rates of morphological evolution were generally low across the phylogeny for both PC1 and PC2 scores, with the exception of the fanfish clade (*Pteraclis* and *Pterycombus*). Fanfish rates were substantially higher than those of all other bramids and *Caristius sp.* for PC1. A similar trend existed for head shape evolution on PC1, with all bramids being characterized by relatively low rates, while fanfishes (notably *Pteraclis*) and *Caristius sp.* were characterized by higher rates of evolution. PC2 showed a different pattern, with body shape evolution appearing to be relatively fast at the base of the clade but slowing within each lineage. Head shape PC2 evolution showed a similar pattern, but with a less dramatic reduction in rates, especially within *Caristius sp.* and *Taractes* clades.

These results are consistent with our integration analysis, and show that rates of evolution in head and body shapes are similar across bramids. Further, they reveal that rates are higher in fanfishes, due to a further elaboration of medial fin morphology, and concomitant shifts in head and body shapes along an evolutionary line of least resistance (Schluter 1996).

### Differences in fanfish anatomy are detectable early in ontogeny

Since developmental processes can impact the emergence of phenotypic novelties, we sought to determine how early exaggerated fins appear during fanfish ontogeny—for example, are they pre-patterned in their fully exaggerated form, or are they elaborated over ontogeny? The results of a phenotypic trajectory analysis revealed significant differences in phenotypic trajectory correlations in *Pterycombus* fanfishes compared with the other bramid genera (*Brama*, *Taractes*, and *Taractichthys*) but no significant difference when compared with the manefish genus, *Caristius* (Table 3). The genera *Brama*, *Taractes*, and *Taractichthys* exhibited no significant difference in phenotypic trajectory correlations from one another, and, of those, only *Brama* was significantly different from *Caristius*.

**Table 3:** Results comparing phenotypic trajectory correlations among genera between juvenile and adult ontogenetic stages

	***Brama***	*Caristius*	*Pterycombus*	*Taractes*	*Taractichthys*
***Brama***		**2.14**	**2.79**	1.09	1.16
***Caristius***	**0.040**		1.64	1.81	1.85
***Pterycombus***	**0.019**	0.072		**2.93**	**1.93**
***Taractes***	0.134	0.061	**0.015**		−0.46
***Taractichthys***	0.122	0.058	**0.049**	0.624	

*Z* scores are above and *P* values are below the diagonal. Bolded *P* values and *z* scores indicate significant differences in mean shapes between species. For the purpose of significance testing, α = 0.05.

**Fig. 7:**
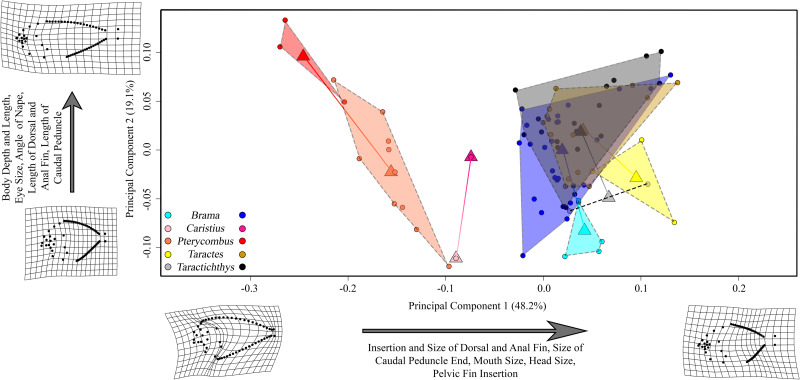
Morphospace of available bramid taxa and a juvenile/adult *Caristius* sample, with trajectories imposed for group means. Lighter colors represent juveniles, darker colors represent adults, and gradient-filled lines indicate the trajectory from juvenile to adult. All bramids, with the exception of fanfishes (here, *Pterycombus* spp.), occupy net neutral and positive scores during both juvenile and adult stages. Fanfishes occupy negative scores. Manefish (*Caristius fasciatus*) occupy an intermediate region of morphospace between fanfishes and all other bramids. Triangles represent mean shape values, while circles represent individual specimens. Circles that exist within a triangle indicate that a single specimen was available for use.

The first two axes of morphospace explained 48.2 and 19.1% of the total variation, respectively (Fig. 7). The first axis can largely be attributed to medial fin insertion points and length, eye size, and the relative ratio of head: body size. It was on this axis that fanfishes are isolated from the other bramid taxa, regardless of ontogenetic stage. The second axis primarily explained body length: depth ratio and eye size, with positive scores relating to smaller eyes and longer bodies. The second axis largely separated the two ontogenetic stages across species, with juvenile groups overwhelmingly characterized by having lower scores than their adult counterparts.

Since it was apparent that fanfish juveniles are morphologically distinct from other bramids, we cleared and stained two larval and one juvenile *Pterycombus brama* and three larval *Brama dussumieri* specimens. Given the substantial emphasis that previous analyses had placed on dorsal fin insertion and relative head size, we focused on identifying skeletal differences in these regions between the species at distinct ontogenetic stages. Larval *P. brama* showed an abundance of dorsal pterygiophores that extended into the caudal neurocranium. They can be seen dorsal to the neurocranium, which consequently lacks a supraoccipital crest (SOC) (Supplemental Figure S3A). At the juvenile stage, *P. brama* still lack a noticeable SOC, as pterygiophores continue to grow just above the posterior neurocranium (Supplemental Figure S3B). Alternatively, *B. dussumieri* possessed a relatively robust SOC by the late larval stage (Supplemental Figure S3C and D), and pterygiophore development was restricted to posterior of the neurocranium.

These data suggest that key aspects of fanfish anatomy are predisposed to accommodate the formation of an expanded dorsal fin, and thus, some of the earliest stages of skeletal development appear to have been altered during the evolution of this trait.

### Fanfish craniofacial architecture suggests co-option of important elements

**Fig. 8:**
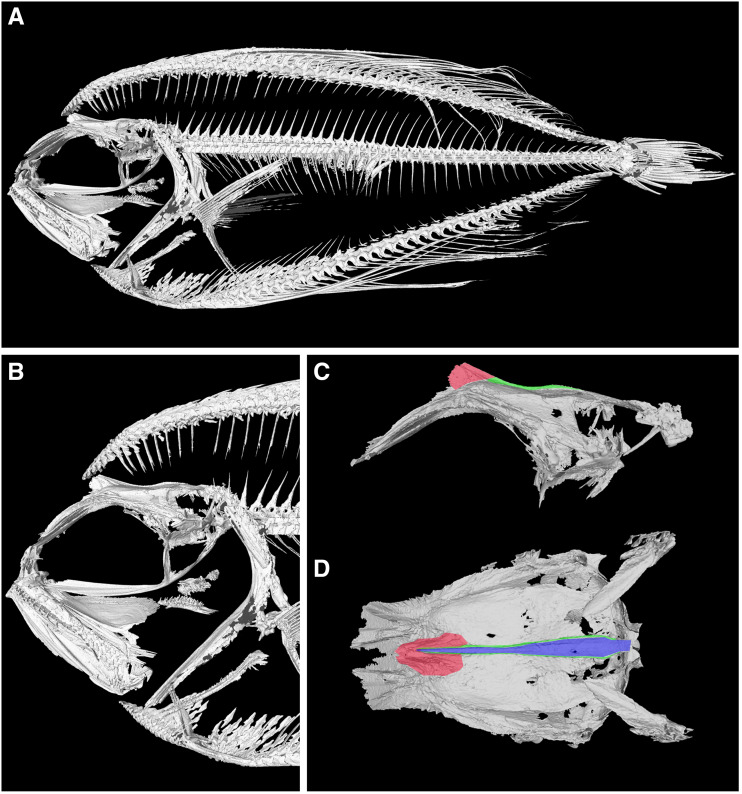
Reconstruction from µCT scans of a representative *Pterycombus petersii*, standard length 7.9 cm. (**A**) High-resolution full body scan. (**B**) Craniofacial skeleton showing internal elements of the dorsal fin. Lateral (**C**) and dorsal (**D**) view of the digitally isolated neurocranium to highlight the substantially altered supraoccipital crest (red), its proximal bifurcation (green), and the deep cleft that accommodates dorsal fin pterygiophores and their associated musculature (blue; not visible in C)

Given the results of our developmental analyses, we wanted to examine in greater resolution the anatomical relationship between the dorsal fin and skull in fanfishes. Accordingly, we gathered µCT data from a single adult *Pterycombus petersii* specimen (Fig. 8A), collected off the coast of Hawaii in the Fall of 2018 from the stomach of a tuna, *Thunnus albacares.* Again, given the emphasis that previous analyses have place on this region, we focused on the craniofacial skeleton (Fig. 8B) to identify osteological elements that may have been altered to accommodate extreme anterior dorsal fin expansion. Consistent with our developmental data, skeletal components of the dorsal fin occupy roughly half the space that would otherwise be available for SOC growth. As a result, the SOC is greatly reduced and restricted to the anterior neurocranium (Fig. 8C). The loss of a posterior SOC may lead to a greatly reduced (relative) area for epaxial muscle attachment, something that we hope to address quantitatively in future studies. In addition, there is a bifurcation of the posterior skull that forms a cleft and appears to be associated with the intruding pterygiophores from the dorsal fin (Fig. 8D).

**Fig. 9:**
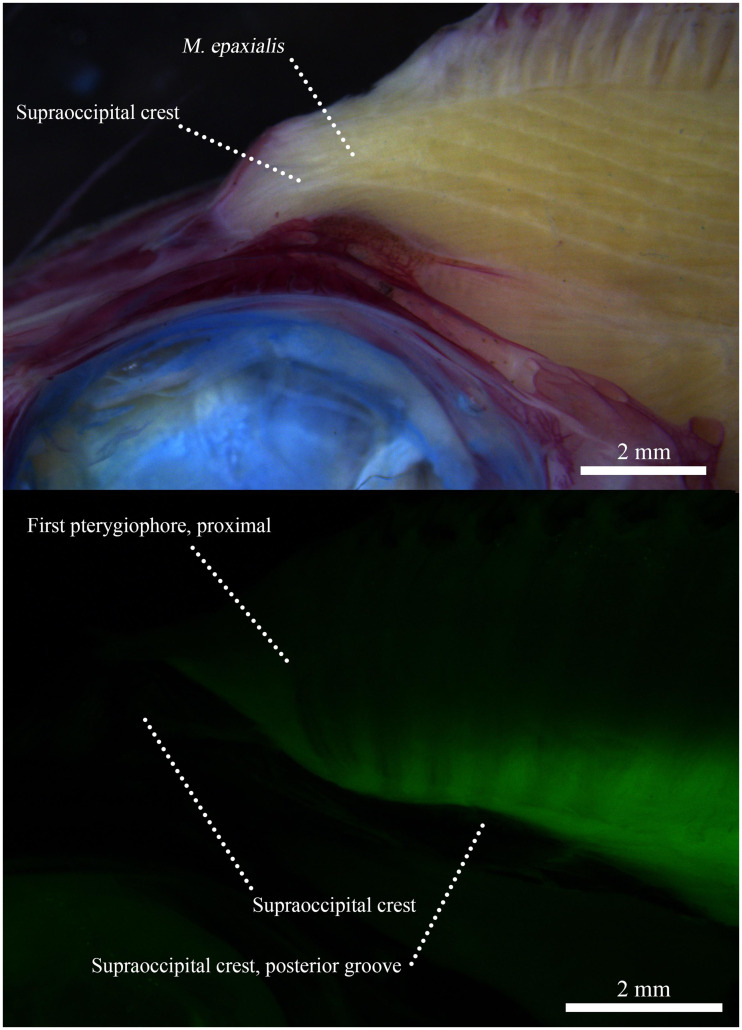
(**A**) Image of alcian and EtOH alizarin stained *Pterycombus petersii*, illustrating epaxial muscle attachment to the supraoccipital crest. (**B**) Same specimen under fluorescent lighting with GFP filter and epaxial musculature removed. Bright green areas represent endogenous illumination, highlighting connective and muscle tissues originating from the dorsal fin skeleton and inserting on the skull posterior to the supraoccipital crest. Black and low contrast areas indicate bone.

Gross dissection of this specimen confirmed a truncated SOC and reduced epaxial muscle attachment (Fig. 9). Further, the posterior bifurcation of the skull appears to accommodate pterygiophore growth and function, as the base of anterior pterygiophores extended to the cleft formed by the bifurcation, which is also the site of attachment for the associated dorsal fin musculature. Dorsal fin musculature is highly complex, comprised of a number of muscles, and detailed myological work will be the topic of future investigation.

Another notable aspect of fanfish anatomy includes a lower jaw that extends posteriorly to the caudal margin of the orbit, which predicts a large gape. Further, the ascending arm of the premaxilla is highly reduced, which likely results in limited, or nonexistent, premaxillae protrusion (Westneat 1990; Cooper and Westneat 2009), and a jaw opening mechanism that is primarily driven by rotation of the articular-quadrate joint. The oral jaw anatomy described here appears ubiquitous within the family Bramidae (Supplemental Figure S4A–C; Supplemental Figure S5A and B) and is similar to what we observe in Caristiidae (Supplemental Figure S4D).

## Discussion

While evolution can yield an incredible number of phenotypic outcomes, only a fraction of those destinations are accessible to any given population due to evolutionary constraints. As adaptive phenotypes evolve along one trajectory, the degree to which related traits (e.g., physiological, developmental, morphological) can diverge and diversify may be limited, canalizing future phenotypic trajectories on an evolutionary scale. Pareto optimality theory echoes this, arguing that no system can be simultaneously improved for all tasks at once and that in order to improve one aspect of a system, a sacrifice, or trade-off, must be made elsewhere (McGhee 2007; Kennedy 2010; Shoval et al. 2012).

Our work on bramids exemplifies not only putative anatomical/morphological constraints (proximate) associated with the development of an exaggerated trait, but also the long-term, evolutionary costs (ultimate) in the sense of constraining future lineages to increasingly fewer adaptive peaks (Conway 2003). Using previously deposited mitochondrial DNA sequences, we create the most speciose bramid tree to date and illustrate genera-specific relationships to the outgroup, Caristiidae (manefishes). While our morphometric data are limited to gross form, we quantitatively demonstrate the substantial differences in overall head and body shape, and rates of morphological evolution in fanfishes compared with their bramid relatives. Through this, we also find support for manefishes being relatively intermediate in overall form to what we identify as two divergent bramid sub-groups, the subfamily Ptericlinae (genera *Pteraclis* and *Pterycombus*) and the remaining bramids (genera *Brama*, *Eumegistus*, *Taractes*, *Taractichthys*, and, presumably, *Xenobrama*). Further, to accommodate the exaggerated fin morphology, we identify and present morphological data that illustrate pronounced modifications to the craniofacial skeleton in the Ptericlinae. Many of these modifications, with the exception of the supraoccipital crest, persist among the other bramids, notably the oral jaw architecture of the other bramid taxa that may contribute to the known and predicted feeding ecology of these fishes.

We set out to understand the anatomical and evolutionary constraints associated with extreme dorsal fin morphology in the family Bramidae. The fanfishes, monophyletic and totaling five of the 20 extant bramid species, stand apart from the rest of the family and demonstrate a greatly exaggerated trait that, we predict, would come at a functional cost (Adriaens and Herrel 2009). We also predict that, if this phenotypic trait was ancestral to the family, there would be a detectable evolutionary cost associated with the other bramid lineages (Farnsworth and Niklas 1995; Tendler et al. 2015). What follows is a discussion of our results in the larger overall context of how extreme adaptations can create functional constraints and how those constraints may influence evolutionary trajectories.

### Exaggerated fin morphology appears ancestral and may constrain foraging anatomy in bramids

The evolutionary relationships within the family Bramidae have been poorly resolved, and the majority of trees include only a small number of bramid taxa (Chen et al. 2016; Liu et al. 2016; Xu et al. 2018). Previous hypotheses suggested that the benthopelagic genus *Eumegsitus* (Bramidae) was the most ancestral (Mead 1972). In his monograph, Mead speculates that the family Caristiidae, (typically represented by deep, robust bodies and exaggerated medial fin morphology), may have derived from a *Pteraclis-*like ancestor (Bellottii 1903). However, our study, as well as recent work presented by Miya et al. (2013) and Friedman et al. (2019), suggests that the family Bramidae diverged from caristiids. This assertion is also supported by the fossil record (Casier 1966; Bannikov and Tyler 1994; Ellison et al. 1994; Baciu and Bannikov 2003; Miya et al. 2013), which dates caristiids prior to bramids. If true, it is possible that exaggerated dorsal fin morphology is the ancestral state for the family Bramidae and that the Ptericlinae continued to exaggerate the extreme medial fin morphology present in manefishes. In this proposed scenario, the last common ancestor to the other bramid lineages likely lost this exaggeration, but maintained, and continued to develop, deeper, robust, body shape morphologies.

While we are able to identify four distinct body shape morphologies within the family Bramidae (Fig. 3; the fusiform body shape of *Taractes*, the deep bodies of *Taractichthys*, the intermediate form of *Brama*, and the elongated *Pteraclis*), the most striking anatomical feature remains the relative proportions of the medial fins. In particular, PC1 explained nearly 63% of the total variation and mainly captured variation in medial fin size and position (e.g., the deformation grid describing extreme shape along this axis nearly folds in on itself at the junction between the head and median fins; Fig. 3). This is again highlighted in ontogenetic trajectories (Fig. 7), where we see a separation of fanfishes from all other bramids along PC1 and noticeable differences in dorsal fin placement early during development. These data suggest that morphological differences between fanfishes and other bramids arise early in development and may therefore be “locked-in.” They suggest further that differences in rates of morphological evolution between these two lineages may be linked to alternate developmental patterning mechanisms with respect to the medial fins. Gaining a better understanding of bramid developmental processes and mechanisms will be a fruitful line of future research.

Across fishes, dorsal fin structure is diverse and functions in myriad tasks, including, but not limited to, locomotion (Breder 1926; Loofbourrow 2006; Jagnandan and Sanford 2013), protection (Hoogland et al. 1956), cutwaters, hydrodynamic efficiency (Drucker and Lauder 2001; Nauen and Lauder 2001; Wang et al. 2020), advertising (Allen and Nicoletto 1997), herding prey (Domenici et al. 2014), generating rapid propulsion and bursts of speed (Gibb et al. 1999; Nauen and Lauder 2001), reducing yaw and roll in fast swimmers (Webb 1984; Weihs 1993; McGowan 1999), and increased maneuverability (Standen and Lauder 2005). For fishes with large, erectable fins, the increased surface area allows for greater deflection of water (Lamb 1975), thereby increasing their ability to change direction. This feature is more common in prey species but can be seen in some predators as well (e.g., Istiophoriformes, *Coryphaena*).

Typically, the dorsal fin begins 3–5 vertebrae caudal to the cranio-vertebral joint following the supraneurals (Thys 1997; Jimenez et al. 2018). This anatomical configuration facilitates cranial elevation during suction feeding, whereby the skull rotates dorsally to facilitate mouth opening, premaxillary protrusion, and hyoid depression (Lauder 1981; Lauder and Liem 1981; Svanbäck et al. 2002; Wainwright et al. 2006; Tegge et al. 2020). Cranial elevation is of great importance to suction-feeding fishes (Carroll and Wainwright 2006; Coughlin and Carroll 2006; Camp and Brainerd 2014; Van Wassenbergh et al. 2015), and thus, the predominant insertion point of the dorsal fin usually begins posterior to three to five free-floating interneural bones. Functionally, this void of articulated bones creates a region of “folding” as the epaxial musculature contracts on the posterior region of a fish’s skull to elevate the neurocranium (Jimenez et al. 2018). Having a dorsal fin attach directly to the top of the skull, as seen in the fanfishes, eliminates this void, which likely compromises the ability of the skull to rotate about the cranio-vertebral joint, a stereotypical feature of suction-feeding. Ram-feeders also exhibit cranial rotation (Bergert and Wainwright 1997; Ferry-Graham et al. 2001; Porter and Motta 2004), but generally to a lesser degree. Rather, this mode of feeding is more strongly associated with long jaws and the ability to generate large gapes (e.g., mackerels, barracuda, etc.) to engulf evading prey (Ferry-Graham, Wainwright, and Bellwood 2001; Ferry-Graham et al. 2001; Porter and Motta 2004). As a whole, the Bramidae possess long lower jaws that are relatively constant in size when regressed against head size (Supplemental 5), consistent with large gapes. Further, long jaws coupled with limited upper jaw protrusion suggests that bramids exhibit a notched, rather than circular, mouth opening, which should compromise suction performance by altering flow dynamics critical to successful suction feeding (Carroll et al. 2004). We suggest that the evolution of a dorsal fin that extends anteriorly into the cranial region and mechanically inhibits skull rotation, while potentially increasing swimming maneuverability, predisposed the lineage toward the ram-feeding end of the ram-suction prey-capture continuum.

In *Pterycombus petersii* (and other fanfishes), we observe a combination of anatomical features consistent with such a trade-off. First, they have extremely large, erectable fins, which suggest they use these fins for evading predators (e.g., tuna) and/or pursuing elusive prey (e.g., cephalopods, myctophids). In addition, they possess modified scales at the base of the medial fins (allowing for complete dorsal and anal fin retraction and concealment), a large aspect ratio of the caudal fin (throughout the family Bramidae and rivaling that of other pelagic cruisers like *Rachycentron canadum*), and symmetrical rows of raised, recurved scale spines (found throughout the family on various bramid species) reminiscent of placoid scales in elasmobranchs known to increase hydrodynamic efficiency (Bechert et al. 1997; Oeffner and Lauder 2012; Wen et al. 2014). Together, these traits suggest that, despite having large, seemingly cumbersome fins, fanfishes have adapted methods for increasing their hydrodynamic efficiency, enabling them to swim at high-cruising speeds, relative to their body size (by retracting their fins and creating an elongate, streamlined body form), and vastly improve their maneuverability when the need arises by erecting their exaggerated fins. The evolution of elaborate fins appears to come with a substantial modification of the occipital region of the skull (Fig. 8), including a considerable reduction of the SOC and a bifurcated cleft in the occipital region where the dorsal fin musculature attaches. This novel anatomical modification demonstrates a direct mechanical linkage between the dorsal fin and the neurocranium. A shortened SOC is also notable, as this bone contributes to the in-lever during the action of cranial elevation (Carroll et al. 2004). A short (nearly absent) SOC in fanfishes should therefore result in a short in-lever and less effective mechanical system for suction feeding. While expanded surface area on top of the neurocranium could mitigate the lack of a SOC in fanfishes, this seems unlikely as skull width is not noticeably greater in fanfishes compared with other bramids.

High-speed filming of open water species is challenging, and the manipulation of fixed specimens (e.g., to directly assess jaw protrusion or cranial elevation) is all but impossible. We therefore make all kinematic inferences about bramid foraging with caution. Despite these difficulties, large museum specimens did allow us to take advantage of preservation state, allowing us to make hypotheses about feeding mechanics. Regarding this, of all specimens examined, we make no observation of any fixed museum specimen showing meaningful premaxilla protrusion, but do observe substantial lower jaw depression (Supplemental Figure 6). Nevertheless, teleosts are an exceptionally well-studied kinematic system, with a detailed understanding of the connection between form and function (for review, see Liem and Osse 1975). Based on the functional anatomy of bramids as a whole, we hypothesize that this lineage is well adapted to move and forage within the open ocean habitat, but is simultaneously constrained to a narrower realm of niche-space (e.g., most likely strict ram-feeders). If true, this would represent an example of how proximate form-function trade-offs may translate to constrained patterns of morphological evolution.

### The influence of historical contingency on bramid ecology and evolution

While the path of evolution is largely unpredictable, future outcomes of a lineage are undoubtedly reliant on the historical states (Gould and Woodruff 1990). In this respect, the numerous small changes that accumulate in lineages create limitations that can render *some* aspects of evolution predictable, or provide a rationale for why certain realms of phenotypic space have not been occupied (Conway 2003). The evolution of the family Bramidae provides an excellent system to explore these ideas.

Events of natural history can be difficult to evaluate and require an adequate record of a lineage’s past to assist in making inferences about the contemporary phenotypes that we observe in living taxa (Gould and Woodruff 1990). However, using fossils in conjunction with molecular data can help inform such predictions. For instance, fossils of caristiid (*Exellia proxima*, *E. velifer*; [Bannikov and Tyler 1994]) and bramid (*Paucaichthys neamtensis*, *P. elamensis*; [Baciu and Bannikov 2003]; [Přikryl and Bannikov 2014]) relatives show striking similarities in both overall body shape, craniofacial anatomy, and medial fin morphology. Our phylogeny and morphometric analyses, along with the recent phylogenies of others (Miya et al. 2013; Friedman et al. 2019), suggest that caristiids are sister to bramids and possess an intermediate form in terms of gross head and body shape (Fig. 4). Taken together, these data suggest that expanded medial fin morphology was ancestral, and therefore early diversification within this lineage occurred within the context of this exaggerated trait.

We find that bramid evolution was initially marked by rapid diversification, followed by a linear decline in speciation rate. One explanation for an initial burst of diversification could be the exploitation of new resources following the extinction of predatory Mesozoic teleosts (Friedman 2009, 2010). An alternate, though not mutually exclusive, hypothesis may involve a shift in locomotor behavior in the bramid stem lineage. For example, while data are scarce, descriptions of caristiid ecology are largely centered around their seemingly poor swimming ability and mysterious relationship to siphonophores (Janssen et al. 1989; Benfield et al. 2009). This is a striking contrast to the predominately open ocean bramids, which anatomically appear well-adapted at attaining high speeds (Legendre 1924) and are known for their substantial migratory habits (Mead 1972). Thus, it is plausible that the initial burst of diversification we detect in bramids is the result of one lineage losing exaggerated fin morphology entirely, opting for maximizing high speeds, navigating the high seas, and growing to much larger sizes and bulk than fanfishes, while the other lineage maintains exaggerated fins, but evolves a functional work-around to poor swimming performance, enabling it to excel at both maneuverability and speed. Notably, however, in both lineages the evolution of craniofacial shape appears to be relatively constrained, which may be due to an ancestral trade-off between the historical locomotion and foraging architecture that has predisposed the bramid lineage toward ram-feeding. In summary, the story of bramid evolution may be one whereby a lineage takes advantage of ecological opportunity (e.g., Mesozoic extinction) by modulating traits that remain highly evolvable (e.g., medial fins), while experiencing niche-space limitations (e.g., to ram-feeding) due to historical constraints.

### Summary and significance

The evolution of novel traits not only introduces new constraints to a system, but must also work within the confines of previous evolutionary constraints (Jacob 1977; Gould and Woodruff 1990; Losos et al. 1998; Blount et al. 2012), thereby limiting future adaptive peaks to an increasingly narrow field of view (Wright 1932; Arnold 1992; Schluter 1996). Nearly 50 years ago, Mead (1972) remarked that the evolution and phylogenetic relationships within family Bramidae deserved further study. Due to the rarity of several species, this has been a challenging task to accomplish; however, recent work has made progress toward clarifying the phylogenetic relationship among bramids, as well as between bramids and other open ocean lineages. Here, we build upon this work to explore the evolution of exaggerated fins, hypothesize putative trade-offs between fin and skull functional morphology, and attempt to identify how these may have shaped bramid evolutionary trajectories. To summarize, given the SOC and intraneural bones of other bramids, they (nonfanfish bramids) should be able to generate suction. However, since their ancestral state likely had extreme dorsal fin morphology, and evolved to maximize ram feeding as a consequence, the evolution of the entire family appears to have been constrained. All in all, we are excited by the prospect that this system offers examples of, and provides insight into, how the development of an exaggerated trait can introduce both proximate and ultimate trade-offs in a lineage.

## Supplementary Material

obab003_Supplementary_Data

## References

[obab003-B1] Adams D , Collyer ML, Kaliontzopoulou A. 2018. Geomorph: software for geometric morphometric analysis.

[obab003-B2] Adams DC. 2014. Quantifying and comparing phylogenetic evolutionary rates for shape and other high-dimensional phenotypic data. Syst Biol 63:166–77. 24335426 10.1093/sysbio/syt105

[obab003-B3] Adams DC , Collyer ML. 2016. On the comparison of the strength of morphological integration across morphometric datasets. Evolution (N Y) 70:2623–31. 10.1111/evo.13045 27592864

[obab003-B4] Adams DC , Collyer ML. 2018. Multivariate phylogenetic comparative methods: evaluations, comparisons, and recommendations. Syst Biol 67:14–31. 28633306 10.1093/sysbio/syx055

[obab003-B5] Adams DC , Collyer ML, Otarola-Castillo E. 2014. R: package, geomorph: software for geometric morphometric analysis (https://github.com/geomorphR/geomorph).

[obab003-B6] Adams DC , Felice RN. 2014. Assessing trait covariation and morphological integration on phylogenies using evolutionary covariance matrices. PLoS One 9:e94335. 24728003 10.1371/journal.pone.0094335 PMC3984176

[obab003-B7] Adriaens D , Herrel A. 2009. Functional consequences of extreme morphologies in the craniate trophic system. Physiol Biochem Zool 82:1–6. 19061414 10.1086/594382

[obab003-B8] Ali AM , McNoon A. 2009. Additions to benthopelagic fish fauna of the Aden Gulf-Arabian Sea (Actinopterygii: Bramidae and Sternoptychidae). J Fish Aquat Sci 5:23–32.

[obab003-B9] Allen JM , Nicoletto PF. 1997. Response of *Betta splendens* to computer animations of males with fins of different length. Copeia 1997:195–9.

[obab003-B10] Arnold SJ. 1983. Morphology, performance and fitness. Integr Comp Biol 23:347–61.

[obab003-B11] Arnold SJ. 1992. Constraints on phenotypic evolution. Am Nat 140:S85–107. 19426028 10.1086/285398

[obab003-B12] Baciu D , Bannikov AF. 2003. *Paucaichthys neamtensis* gen. et sp. nova-the first discovery of sea breams (Bramidae) in the Oligocene of Romania. Vopr ikhtiologii 43:598–602.

[obab003-B13] Bannikov AF. 2012. The first record of the genus *Isurichthys* (Perciformes, Ariommatidae) in the Lower Oligocene of the Northern Caucasus. Paleontol J 46:171–6.

[obab003-B14] Bannikov AF , Tyler JC. 1994. A revision of the Eocene fish family Exelliidae (Perciformes). Paleontol J 28:128–40.

[obab003-B15] Bechert DW , Bruse M, Hage W, Van Der Hoeven JGT, Hoppe G. 1997. Experiments on drag-reducing surfaces and their optimization with an adjustable geometry. J Fluid Mech 338:59–87.

[obab003-B16] Bellottii C. 1903. Di un nuovo pteraclide giapponese. Atti della Soc Ital di Sci Nat di Milano 42:136–9.

[obab003-B17] Benfield MC , Caruso JH, Sulak KJ. 2009. *In situ* video observations of two manefishes (Perciformes: Caristiidae) in the Mesopelagic zone of the northern Gulf of Mexico. Copeia 2009:637–41.

[obab003-B18] Benson DA , Cavanaugh M, Clark K, Karsch-Mizrachi I, Lipman DJ, Ostell J, Sayers EW. 2013. GenBank. Nucleic Acids Res 41:36–42. 10.1093/nar/gks1195 PMC3531190 23193287

[obab003-B19] Bergert BA , Wainwright PC. 1997. Morphology and kinematics of prey capture in the syngnathid fishes *Hippocampus erectus* and *Syngnathus floridae*. Mar Biol 127:563–70.

[obab003-B20] Blount ZD , Barrick JE, Davidson CJ, Lenski RE. 2012. Genomic analysis of a key innovation in an experimental *Escherichia coli* population. Nature 489:513–8. 22992527 10.1038/nature11514 PMC3461117

[obab003-B21] Bouckaert R , Heled J, Kühnert D, Vaughan T, Wu CH, Xie D, Suchard MA, Rambaut A, Drummond AJ. 2014. BEAST 2: a software platform for Bayesian evolutionary analysis. PLoS Comput Biol 10:e1003537–6. 24722319 10.1371/journal.pcbi.1003537 PMC3985171

[obab003-B22] Bouckaert RR , Drummond AJ. 2017. bModelTest: Bayesian phylogenetic site model averaging and model comparison. BMC Evol Biol 17:11. 28166715 10.1186/s12862-017-0890-6 PMC5294809

[obab003-B23] Breder C. Jr 1926. The locomotion of fishes. Zoologica 4:159–291.

[obab003-B24] Brodie ED III , Brodie ED. Jr 1999. Costs of exploiting poisonous prey: evolutionary trade-offs in a predator-prey arms race. Evolution (N Y) 53:626–31. 10.1111/j.1558-5646.1999.tb03798.x 28565425

[obab003-B25] Camp AL , Brainerd EL. 2014. Role of axial muscles in powering mouth expansion during suction feeding in largemouth bass (Micropterus salmoides). J Exp Biol 217:1333–45. 24363416 10.1242/jeb.095810

[obab003-B26] Carroll AM , Wainwright PC. 2006. Muscle function and power output during suction feeding in largemouth bass, Micropterus salmoides. Comp Biochem Physiol - A Mol Integr Physiol 143:389–99. 16458031 10.1016/j.cbpa.2005.12.022

[obab003-B27] Carroll AM , Wainwright PC, Huskey SH, Collar DC, Turingan RG. 2004. Morphology predicts suction feeding performance in centrarchid fishes. J Exp Biol 207:3873–81. 15472018 10.1242/jeb.01227

[obab003-B28] Carvalho-filho A , Marcovaldi G, Sampaio CLS, Paiva MIG, Duarte LAG. 2009. First report of rare pomfrets (Teleostei: Bramidae) from Brazilian waters, with a key to Western Atlantic species. Zootaxa 2290:1–26.

[obab003-B29] Casier E. 1966. Faune ichthyologique du London Clay. London Trust Br Museum (Natural Hist London.

[obab003-B30] Charnov EL. 1989. Phenotypic evolution under Fisher’s fundamental theorem of natural selection. Heredity (Edinb) 62:113–6. 2732081 10.1038/hdy.1989.15

[obab003-B31] Chen F , Ma H, Ma C, Zhang H, Zhao M, Meng Y, Wei H, Ma L. 2016. Sequencing and characterization of mitochondrial DNA genome for *Brama japonica* (Perciformes: Bramidae) with phylogenetic consideration. Biochem Syst Ecol 68:109–18.

[obab003-B32] Cignoni P , Callieri M, Corsini M, Dellepiane M, Ganovelli F, Ranzuglia G. 2008. MeshLab: An open-source mesh processing tool. 6th Eurographics Ital Chapter Conf 2008 – Proc, 129–36.

[obab003-B33] Collyer M , Adams D. 2013. Phenotypic trajectory analysis: comparison of shape change patterns in evolution and ecology. Hystrix 24:75–83.

[obab003-B34] Collyer ML , Adams DC. 2018. RRPP : an r package for fitting linear models to high-dimensional data using residual randomization. Methods Ecol Evol 9:1772–9.

[obab003-B35] Collyer ML , Sekora DJ, Adams DC. 2015. A method for analysis of phenotypic change for phenotypes described by high-dimensional data. Heredity 115:357–65. 25204302 10.1038/hdy.2014.75 PMC4815463

[obab003-B36] Conway MS. 2003. Life’s solution. Cambridge: Cambridge University Press.

[obab003-B37] Cooper WJ , Westneat MW. 2009. Form and function of damselfish skulls: rapid and repeated evolution into a limited number of trophic niches. BMC Evol Biol 9:24–17. 19183467 10.1186/1471-2148-9-24 PMC2654721

[obab003-B38] Coughlin DJ , Carroll AM. 2006. In vitro estimates of power output by epaxial muscle during feeding in largemouth bass. Comp Biochem Physiol - A Mol Integr Physiol 145:533–9. 17029993 10.1016/j.cbpa.2006.08.026

[obab003-B39] Darwin C. 1859. On the origin of species.

[obab003-B40] Datovo A , Vari RP. 2013. The jaw adductor muscle complex in Teleostean fishes: evolution, homologies and revised nomenclature (Osteichthyes: Actinopterygii). PLoS One 8:e60846. 23565279 10.1371/journal.pone.0060846 PMC3614958

[obab003-B41] Denton JSS , Adams DC. 2015. A new phylogenetic test for comparing multiple high-dimensional evolutionary rates suggests interplay of evolutionary rates and modularity in lanternfishes (Myctophiformes; Myctophidae). Evolution (N Y) 69:2425–40. 10.1111/evo.12743 26278586

[obab003-B42] Domenici P , Wilson ADM, Kurvers RHJM, Marras S, Herbert-Read JE, Steffensen JF, Krause S, Viblanc PE, Couillaud P, Krause J. 2014. How sailfish use their bills to capture schooling prey. Proc R Soc B Biol Sci 281:1–6. 10.1098/rspb.2014.0444 PMC4043100 24759865

[obab003-B43] Drucker EG , Lauder GV. 2001. Locomotor function of the dorsal fin in teleost fishes: experimental analysis of wake forces in sunfish. J Exp Biol 204:2943–58. 11551984 10.1242/jeb.204.17.2943

[obab003-B44] Drummond AJ , Ho SYW, Phillips MJ, Rambaut A. 2006. Relaxed phylogenetics and dating with confidence. PLoS Biol 4:e88–710. 16683862 10.1371/journal.pbio.0040088 PMC1395354

[obab003-B45] Ellison R , Knox R, Jolley D, King C. 1994. A revision of the lithostratigraphical classification of the early Palaeogene strata of the London Basin and East Anglia. Proc Geol Assoc 105:187–97.

[obab003-B46] Farnsworth KD , Niklas KJ. 1995. Theories of optimization, form and function in branching architecture in plants. Funct Ecol 9:355–63.

[obab003-B47] Ferry-Graham LA , Wainwright PC, Bellwood DR. 2001. Prey capture in long-jawed butterflyfishes (Chaetodontidae): the functional basis of novel feieding habits. J Exp Mar Bio Ecol 256:167–84. 11164861 10.1016/s0022-0981(00)00312-9

[obab003-B48] Ferry-Graham LA , Wainwright PC, Darrin Hulsey C, Bellwood DR. 2001. Evolution and mechanics of long jaws in butterflyfishes (Family Chaetodontidae). J Morphol 248:120–43. 11304744 10.1002/jmor.1024

[obab003-B49] Fierstine H , Huddleston R, Takeuchi G. 2012. Neogene bony fishes of California: a systematic inventory of all published accounts. Calif Acad Sci 1:208.

[obab003-B50] Fitzjohn RG. 2012. Diversitree: comparative phylogenetic analyses of diversification in R. Methods Ecol Evol 3:1084–92.

[obab003-B51] Friedman M. 2009. Ecomorphological selectivity among marine teleost fishes during the end-Cretaceous extinction. Proc Natl Acad Sci U S A 106:5218–23. 19276106 10.1073/pnas.0808468106 PMC2664034

[obab003-B52] Friedman M. 2010. Explosive morphological diversification of spiny-finned teleost fishes in the aftermath of the end-Cretaceous extinction. Proc R Soc B Biol Sci 277:1675–83. 10.1098/rspb.2009.2177 PMC2871855 20133356

[obab003-B53] Friedman M , Feilich KL, Beckett HT, Alfaro ME, Faircloth BC, Černý D, Miya M, Near TJ, Harrington RC. 2019. A phylogenomic framework for pelagiarian fishes (Acanthomorpha: Percomorpha) highlights mosaic radiation in the open ocean. Proc R Soc B Biol Sci 286:20191502. 10.1098/rspb.2019.1502 PMC6742994 31506051

[obab003-B54] Gibb AC , Dickson KA, Lauder GV. 1999. Tail kinematics of the chub mackerel *Scomber japonicus*: testing the homocercal tail model of fish propulsion. J Exp Biol 202:2433–47. 10460731 10.1242/jeb.202.18.2433

[obab003-B55] González-lorenzo G , González-jiménez JF, Brito A, González JF. 2013. The family Bramidae (Perciformes) from the Canary Islands (Northeastern Atlantic Ocean), with three new records. Cybium 37:295–303.

[obab003-B56] Goodall C. 1991. Procrustes methods in the statistical analysis of shape. J R Stat Soc 53:285–339.

[obab003-B57] Gosline WA. 1971. Functional morphology and classification of teleostean fishes. Honolulu: University Press of Hawaii.

[obab003-B58] Gould SJ , Woodruff DS. 1990. History as a cause of area effects: an illustration from *Cerion* on Great Inagua. Bahamas. Biol J Linn Soc 40:67–98.

[obab003-B59] Gutiérrez E , Fernandez A, Hernández R. 2005. *Brama caribbea* (Pisces: Bramidae), un nuevo registro para las aguas cubanas. Solenodon 5:78–9.

[obab003-B60] Herrel A , Bonneaud C. 2012. Trade-offs between burst performance and maximal exertion capacity in a wild amphibian, *Xenopus tropicalis*. J Exp Biol 215:3106–11. 22660787 10.1242/jeb.072090

[obab003-B61] Herrel A , Podos J, Vanhooydonck B, Hendry AP. 2009. Force-velocity trade-off in Darwin’s finch jaw function: a biomechanical basis for ecological speciation? Funct Ecol 23:119–25.

[obab003-B62] Holzman R , Collar DC, Price SA, Darrin Hulsey C, Thomson RC, Wainwright PC. 2012. Biomechanical trade-offs bias rates of evolution in the feeding apparatus of fishes. Proc R Soc B Biol Sci 279:1287–92. 10.1098/rspb.2011.1838 PMC3282375 21993506

[obab003-B63] Hoogland R , Morris D, Tinbergen N. 1956. The spines of sticklebacks (*Gasterosteus* and *Pygosteus*) as means of defence against predators (*Perca* and *Esox*). Behaviour 10:205–36.

[obab003-B64] Jacob F. 1977. Evolution and Tinkering. Science (80-) 196:1161–6. 10.1126/science.860134 860134

[obab003-B65] Jagnandan K , Sanford CP. 2013. Kinematics of ribbon-fin locomotion in the bowfin, *Amia calva*. J Exp Zool Part A Ecol Genet Physiol 319:569–83. 10.1002/jez.1819 24039242

[obab003-B66] Janssen J , Gibbs RH, Pugh PR. 1989. Association of *Caristius sp.* (Pisces : Caristiidae) with a Siphonophore, *Bathyphysa conifera*. Copeia 1989:198–201.

[obab003-B67] Jawad LA , Al-Mamry JM, Al-Busaidi HK. 2014. New record of the keeltail pomfret, *Taractes rubescens* (Jordan & Evermann, 1887) (Perciformes : Bramidae) from the Sea of Oman. Int J Mar Sci 4:227––30.

[obab003-B68] Jimenez YE , Camp AL, Grindall JD, Brainerd EL. 2018. Axial morphology and 3D neurocranial kinematics in suction-feeding fishes. Biol Open 7. 10.1242/bio.036335 PMC6176947 30237249

[obab003-B69] Kennedy MC. 2010. Functional-structural models optimize the placement of foliage units for multiple whole-canopy functions. Ecol Res 25:723–32.

[obab003-B70] Klingenberg CP. 2011. MorphoJ: an integrated software package for geometric morphometrics. Mol Ecol Resour 11:353–7. 21429143 10.1111/j.1755-0998.2010.02924.x

[obab003-B71] Konuma J , Chiba S. 2007. Trade-offs between force and fit: Extreme morphologies associated with feeding behavior in carabid beetles. Am Nat 170:90–100. 17853994 10.1086/518182

[obab003-B72] Lamb H. 1975. Hydrodynamics. New York: Dover.

[obab003-B73] Langerhans RB , Layman CA, DeWitt TJ. 2005. Male genital size reflects a tradeoff between attracting mates and avoiding predators in two live-bearing fish species. Proc Natl Acad Sci U S A 102:7618–23. 15894618 10.1073/pnas.0500935102 PMC1140428

[obab003-B74] Larsson A. 2014. AliView: a fast and lightweight alignment viewer and editor for large datasets. Bioinformatics 30:3276–8. 25095880 10.1093/bioinformatics/btu531 PMC4221126

[obab003-B75] Lauder GV , Liem KF. 1981. Prey capture by *Luciocephalus pulcher*: implications for models of jaw protrusion in teleost fishes. Environ Biol Fishes 6:257–68.

[obab003-B76] Lauder GV. 1981. Intraspecific functional repertoires in the feeding mechanism of the characoid fishes *Lebiasina, Hoplias* and *Chalceus*. Copeia 1981:154–68.

[obab003-B77] Lee J , Lee W, Kim J. 2019. First reliable record of the keeltail pomfret *Taractes rubescens* (Bramidae : Perciformes) from Korea. Korean J Fish Aquat Sci 52:283–7.

[obab003-B78] Lee WJ , Kim JK. 2015. New record of *Brama dussumieri* (Pisces: Bramidae) from Korea, as revealed by morphological and molecular analyses. Fish Aquat Sci 18:311–6.

[obab003-B79] Legendre R. 1924. *Brama raii* Bl.: sa présence au large des côtes sud de la Bretagne. Bull la Société Zool Fr 49:218–25.

[obab003-B80] Lenov Y. 1998. Late Eocene-Early Oligocene geological and biotical events on the territory of the former Soviet Union. Part II. The geological and biotical events. Moscow: GEOS.

[obab003-B81] Leroi AM , Kim SB, Rose MR. 1994. The evolution of phenotypic life-history trade-offs: an experimental study using *Drosophila melanogaster*. Am Nat 144:661–76.

[obab003-B82] Liem KF , Osse J. 1975. Biological versatility, evolution, and food resource exploitation in African cichlid fishes. Am Zool 15:427–54.

[obab003-B83] Liu X , Tian S, Li W, Wu F, Dai X. 2016. Complete mitochondrial genome of the *Taractes rubescens* (Perciformes: Bramidae). Mitochondrial DNA 27:2809–10. 26258509 10.3109/19401736.2015.1053078

[obab003-B84] Lobo C , Erzini K. 2001. Age and growth of Ray’s bream (*Brama brama*) from the south of Portugal. Fish Res 51:343–7.

[obab003-B85] Loofbourrow H. 2006. Hydrodynamics of balistiform swimming in the picasso triggerfish, *Rhinecanthus aculeatus* [Doctoral dissertation]. University of British Columbia.

[obab003-B86] Losos JB , Jackman TR, Larson A, De Queiroz K, Rodríguez-Schettino L. 1998. Contingency and determinism in replicated adaptive radiations of island lizards. Science (80-) 279:2115–8. 10.1126/science.279.5359.2115 9516114

[obab003-B87] McGhee GR. 2007. The Geometry of evolution: adaptive landscapes and theoretical morphospaces. Cambridge: Cambridge University Press.

[obab003-B88] McGowan C. 1999. A practical guide to vertebrate mechanics. Cambridge: Cambridge University Press.

[obab003-B89] Mead GW. 1972. Bramidae. The Carlsberg Foundation’s oceanographical expedition round the World 1928-30 and previous Dana-expeditions. Dana-Report.

[obab003-B90] Miller MA , Pfeiffer W, Schwartz T. 2010. Creating the CIPRES Science Gateway for inference of large phylogenetic trees. 2010 Gatew Comput Environ Work GCE 2010 1–8.

[obab003-B91] Miya M , Friedman M, Satoh TP, Takeshima H, Sado T, Iwasaki W, Yamanoue Y, Nakatani M, Mabuchi K, Inoue JG, Poulsen JY, Fukunaga T, Sato Y, Nishida M. 2013. Evolutionary origin of the Scombridae (tunas and mackerels): members of a Paleogene adaptive radiation with 14 other Pelagic fish families. PLoS One 8:e73535. 24023883 10.1371/journal.pone.0073535 PMC3762723

[obab003-B92] Moteki M , Arai M, Tsuchiya K, Okamoto H. 2001. Composition of piscine prey in the diet of large pelagic fish in the eastern tropical Pacific Ocean. Fish Sci 67:1063–74.

[obab003-B93] Nauen JC , Lauder GV. 2001. Locomotion in scombrid fishes: visualization of flow around the caudal peduncle and finlets of the chub mackerel *Scomber japonicus*. J Exp Biol 204:2251–63. 11507109 10.1242/jeb.204.13.2251

[obab003-B94] Oeffner J , Lauder GV. 2012. The hydrodynamic function of shark skin and two biomimetic applications. J Exp Biol 215:785–95. 22323201 10.1242/jeb.063040

[obab003-B95] Olsen A , Westneat M. 2015. StereoMorph: an R package for the collection of 3D landmarks and curves using a stereo camera set-up. Methods Ecol Evol 6:351–6.

[obab003-B96] Orr JW , Tuttle V, Donovan C. 2018. *Pterycombus petersii* (Bramidae: Teleostei): first record for the eastern North Pacific. Northwest Nat 99:236–8.

[obab003-B97] Park JH , Kim JK, Moon JH, Kim CB. 2007. Three unrecorded marine fish species from Korean waters. Ocean Sci J 42:231–40.

[obab003-B98] Patek SN , Oakley TH. 2003. Comparative tests of evolutionary trade-offs in a palinurid lobster acoustic system. Evolution (N Y) 57:2082–100. 10.1111/j.0014-3820.2003.tb00387.x 14575329

[obab003-B99] Pelegrin N , Mesquita DO, Albinati P, Caldas FLS, de Queiroga Cavalcanti LB, Costa TB, Falico DA, Galdino JYA, Tucker DB, Garda AA. 2017. Extreme specialization to rocky habitats in Tropidurus lizards from Brazil: trade-offs between a fitted ecomorph and autoecology in a harsh environment. Austral Ecol 42:677–89.

[obab003-B100] Porter HT , Motta PJ. 2004. A comparison of strike and prey capture kinematics of three species of piscivorous fishes: Florida gar (*Lepisosteus platyrhincus*), redfin needlefish (*Strongylura notata*), and great barracuda (*Sphyraena barracuda*). Mar Biol 145:989–1000.

[obab003-B101] Přikryl T , Bannikov AF. 2014. A new species of the Oligocene pomfret fish *Paucaichthys* (Perciformes; Bramidae) from Iran. Neues Jahrb für Geol und Paläontologie - Abhandlungen 272:325–30.

[obab003-B102] Pybus OG , Harvey PH. 2000. Testing macro-evolutionary models using incomplete molecular phylogenies. Proc R Soc B Biol Sci 267:2267–72. 10.1098/rspb.2000.1278 PMC1690817 11413642

[obab003-B103] R Core Team. 2018. R: A language and environment for statistical computing. Vienna, Austria: R A Lang Environ Stat Comput.

[obab003-B104] Rabosky DL. 2006. Likelihood methods for detecting temporal shifts in diversification rates. Evolution (N Y) 60:1152–64. 16892966

[obab003-B105] Rabosky DL. 2014. Automatic detection of key innovations, rate shifts, and diversity-dependence on phylogenetic trees. PLoS One 9:e89543. 24586858 10.1371/journal.pone.0089543 PMC3935878

[obab003-B106] Rabosky DL , Grundler M, Anderson C, Title P, Shi JJ, Brown JW, Huang H, Larson JG. 2014. BAMMtools: an R package for the analysis of evolutionary dynamics on phylogenetic trees. Methods Ecol Evol 5:701–7.

[obab003-B107] Rabosky DL , Lovette IJ. 2008. Density-dependent diversification in North American wood warblers. Proc R Soc B Biol Sci 275:2363–71. 10.1098/rspb.2008.0630 PMC2603228 18611849

[obab003-B108] Rabosky DL , Santini F, Eastman J, Smith SA, Sidlauskas B, Chang J, Alfaro ME. 2013. Rates of speciation and morphological evolution are correlated across the largest vertebrate radiation. Nat Commun 4:1–8. 10.1038/ncomms2958 23739623

[obab003-B109] Rahangdale S , Kumar R, Roul SK, Kannan K, Suresh Kumar K, Ranjith L, Manoj Kumar PP. 2019. Filling missing links in bramids distribution along the Indian coast with first record of big tooth pomfret, *Brama orcini* (Perciformes: Bramidae) from the east coast of India. Indian J Geo-Marine Sci 48:654–61.

[obab003-B110] Rambaut A , Drummond AJ, Xie D, Baele G, Suchard MA. 2018. Posterior summarization in Bayesian phylogenetics using Tracer 1.7. Syst Biol 67:901–4. 29718447 10.1093/sysbio/syy032 PMC6101584

[obab003-B111] Revell LJ. 2012. phytools: an R package for phylogenetic comparative biology (and other things). Methods Ecol Evol 3:217–23.

[obab003-B112] Roff DA , Fairbairn DJ. 2007. The evolution of trade-offs: where are we? J Evol Biol 20:433–47. 17305809 10.1111/j.1420-9101.2006.01255.x

[obab003-B113] Rohlf FJ , Corti M. 2000. Use of two-block partial least-squares to study covariation in shape. Syst Biol 49:740–53. 12116437 10.1080/106351500750049806

[obab003-B114] Schluter D. 1996. Adaptive radiation along genetic lines of least resistance. Evolution (N Y) 50:1766. 10.1111/j.1558-5646.1996.tb03563.x 28565589

[obab003-B115] Sheftel H , Shoval O, Mayo A, Alon U. 2013. The geometry of the Pareto front in biological phenotype space. Ecol Evol 3:1471–83. 23789060 10.1002/ece3.528 PMC3686184

[obab003-B116] Shoval O , Sheftel H, Shinar G, Hart Y, Ramote O, Mayo A, Dekel E, Kavanagh K, Alon U. 2012. Evolutionary trade-offs, pareto optimality, and the geometry of phenotype space. Science (80-) 336:1157–61. 10.1126/science.1217405 22539553

[obab003-B117] Standen EM , Lauder GV. 2005. Dorsal and anal fin function in bluegill sunfish *Lepomis macrochirus*: three-dimensional kinematics during propulsion and maneuvering. J Exp Biol 208:2753–63. 16000544 10.1242/jeb.01706

[obab003-B118] Stearns SC. 1989. Trade-offs in life-history evolution. Funct Ecol 3:259–68.

[obab003-B119] Svanbäck R , Wainwright PC, Ferry-Graham LA. 2002. Linking cranial kinematics, buccal pressure, and suction feeding performance in largemouth bass. Physiol Biochem Zool 75:532–43. 12601610 10.1086/344495

[obab003-B120] Tegge S , Hall J, Huskey S. 2020. Spatial and temporal changes in buccal pressure during prey-capture in the trumpetfish (*Aulostomus maculatus*). Zoomorphology 139:85–95.

[obab003-B121] Tendler A , Mayo A, Alon U. 2015. Evolutionary tradeoffs, pareto optimality and the morphology of ammonite shells. BMC Syst Biol 9:1–12. 25884468 10.1186/s12918-015-0149-z PMC4404009

[obab003-B122] Thys T. 1997. Spatial variation in epaxial muscle activity during prey strike in largemouth bass (Micropterus salmoides). J Exp Biol 200:3021–31. 9359891 10.1242/jeb.200.23.3021

[obab003-B123] Toro E , Herrel A, Irschick D. 2004. The evolution of jumping performance in Caribbean Anolis lizards: solutions to biomechanical trade-offs. Am Nat 164. 10.1086/386347 15266382

[obab003-B124] Van Wassenbergh S , Day SW, Hernández LP, Higham TE, Skorczewski T. 2015. Suction power output and the inertial cost of rotating the neurocranium to generate suction in fish. J Theor Biol 372:159–67. 25769945 10.1016/j.jtbi.2015.03.001

[obab003-B125] Wainwright PC , Huskey SH, Turingan RG, Carroll AM. 2006. Ontogeny of suction feeding capacity in snook, *Centropomus undecimalis*. J Exp Zool 305A:246–52. 10.1002/jez.a.255 16432887

[obab003-B126] Waltzek TB , Wainwright PC. 2003. Functional morphology of extreme jaw protrusion in Neotropical cichlids. J Morphol 257:96–106. 12740901 10.1002/jmor.10111

[obab003-B127] Wang J , Wainwright DK, Lindengren RE, Lauder GV, Dong H. 2020. Tuna locomotion: a computational hydrodynamic analysis of finlet function. J R Soc Interface 17:20190590. 32264740 10.1098/rsif.2019.0590 PMC7211474

[obab003-B128] Webb PW. 1984. Form and function in fish swimming. Sci Am 251:72––83.

[obab003-B129] Weihs D. 1993. Stability of aquatic animal locomotion. Contemp Math 141:443–61.

[obab003-B130] Wen L , Weaver JC, Lauder GV. 2014. Biomimetic shark skin: design, fabrication and hydrodynamic function. J Exp Biol 217:1656–66. 24829323 10.1242/jeb.097097

[obab003-B131] Westneat MW. 1990. Feeding mechanics of teleost fishes (Labridae; Perciformes) – a test of 4-bar linkage models. J Morphol 205:269–95. 29865760 10.1002/jmor.1052050304

[obab003-B132] Westneat MW. 2004. Evolution of levers and linkages in the feeding mechanisms of fishes. Integr Comp Biol 44:378–89. 21676723 10.1093/icb/44.5.378

[obab003-B133] Wright S. 1932. The roles of mutation, inbreeding, crossbreeding and selection in evolution. Sixth Int Congr Genet.

[obab003-B134] Xu L , Wang X, Li H, Du F. 2018. The complete mitochondrial genome of Perciformes fish (*Brama dussumieri*) from South China Sea. Mitochondrial DNA Part B Resour 3:874–7. 10.1080/23802359.2018.1501293 PMC7800298 33490542

[obab003-B135] Yatsu A , Nakamura I. 1989. *Xenobrama microlepis*, a new genus and species of bramid fish, from the Subantarctic waters of the South Pacific. Jpn J Ichthyol 36:190–5.

