# Supplementary material for: Extreme Morphology, Functional Trade-offs, and Evolutionary Dynamics in a Clade of Open-Ocean Fishes (Perciformes: Bramidae)
Source: Integr Org Biol. 2021 Feb 16;3(1):obab003. doi: 10.1093/iob/obab003 (PMC8077895; doi:10.1093/iob/obab003)
Supplement: obab003_Supplementary_Data [file obab003_supplementary_data.pdf]

Supplemental Tables and Figures

Supplemental Table 1. Accession numbers for mitochondrial gene data retrieved from GenBank.

Species	COI	CytB
Brama australisA	KT455496.1	FJ435626.1
Brama australisB	KT455495.1	FJ435625.1
Brama australisC		FJ435624.1
Brama bramaA	HQ564257.1	DQ197933.1
Brama bramaB	HQ564256.1	
Brama dussumieriA	MH638791.1	
Brama dussumieriB	MH638770.1	
Brama japonicaA	MH638780.1	AY973048.1
Brama japonicaB	MH638779.1	KF546812.1
Brama japonicaC		KF546811.1
Brama japonicaD		KF546810.1
Brama japonicaE		KF546809.1
Brama orciniA	JN312891.1	
Brama orciniB	KY371231.1	
Caristius fasciatusA	KY033875.1	
Caristius fasciatusB	KU176441.1	
Caristius macropus	AP005999.1	AP005999.1
Eumegistus illustrisA	KU943868.1	
Eumegistus illustrisB	AP012497.1	AP012497.1
Peprilus paruA	MH378570.1	
Peprilus paruB	MH378544.1	
Peprilus paruC	MH378516.1	
Peprilus simillimusA	KY570356.1	FJ264431.1
Peprilus simillimusB	KY570355.1	FJ264297.1
Peprilus simillimusC	KT247733.1	FJ264296.1
Peprilus triacanthusA	KF930245.1	
Peprilus triacanthusB	KC015786.1	
Peprilus triacanthusC	KC015784.1	
Peprilus triacanthusD	KC015782.1	
Peprilus triacanthusE	NC_022502.1	NC_022502.1
Peprilus triacanthusF	AP012518.1	AP012518.1
Platyberyx sp	HQ564263.1	
Pteraclis aesticolaA	KU892878.1	
Pteraclis aesticolaB	NC_022487.1	NC_022487.1
Pteraclis aesticolaC	AP012499.1	AP012499.1
Pterycombus bramaA	GU225016.1	
Pterycombus bramaB	GU225017.1	
Pterycombus bramaC	GU225018.1	
Pterycombus bramaD	GU225019.1	
Pterycombus petersiiA	HQ564259.1	
Pterycombus petersiiB	HQ564260.1	
Taractes asperA	GU440550.1	AY973049.1
Taractes asperB	AP012498.1	AP012498.1
Taractes asperC	NC_022486.1	NC_022486.1
Taractes rubescensA	HQ564480.1	
Taractes rubescensB	KY372191.1	

Taractes rubescensC	KY372190.1	
Taractes rubescensD	NC_028079.1	NC_028079.1
Taractes rubescensE	KR349364.1	KR349364.1
Taractichthys longipinnisA	EF609476.1	EF392625.1
Taractichthys longipinnisB	AB639845.1	
Taractichthys steindachneriA	KY372198.1	
Taractichthys steindachneriB	KY372197.1	
Taractichthys steindachneriC	KY372196.1	
Taractichthys steindachneriD	EF609477.1	
Taractichthys steindachneriE	NC_027858.1	NC_027858.1
Taractichthys steindachneriF	KT153629.1	KT153629.1
Xenobrama microlepisA	KX497161.1	
Xenobrama microlepisB	EF609495.1	

Supplemental Table 2. Museum prefixes and lot number for each specimen used in morphometric analyses. Museum prefixes: **MCZ** Museum of Comparative Zoology, Harvard College, **USNM** National Museum of Natural History, **AUNMH** Australian Museum of Natural History.

Lot Number	Species	Life Stage
MCZ_Lot_40826_1	*Brama brama*	Juvenile
MCZ_Lot_40826_2	*Brama brama*	Juvenile
MCZ_Lot_40826_3	*Brama brama*	Juvenile
MCZ_Lot_40826_4	*Brama brama*	Juvenile
MCZ_Lot_40826_5	*Brama brama*	Juvenile
MCZ_Lot_46336	*Brama brama*	Adult
MCZ_Lot_46337	*Brama brama*	Adult
MCZ_uncatalogued	*Brama brama*	Adult
MCZ_148287	*Brama dussumieri*	Adult
MCZ_148287_2	*Brama dussumieri*	Adult
MCZ_Lot_44137	*Brama japonica*	Adult
MCZ_Lot_44138	*Brama japonica*	Adult
MCZ_Lot_44142	*Brama japonica*	Adult
MCZ_Lot_44143	*Brama japonica*	Adult
MCZ_Lot_44144	*Brama japonica*	Adult
MCZ_Lot_46327	*Brama japonica*	Adult
MCZ_Lot_46333	*Brama japonica*	Adult
MCZ_Lot_46334	*Brama japonica*	Adult
MCZ_Lot_46335	*Brama japonica*	Adult
MCZ_Lot_46339	*Brama japonica*	Adult
MCZ_Lot_46340	*Brama japonica*	Adult
MCZ_Lot_46341	*Brama japonica*	Adult
MCZ_Lot_46342	*Brama japonica*	Adult
MCZ_Lot_46343	*Brama japonica*	Adult
MCZ_Lot_46344	*Brama japonica*	Adult
MCZ_Lot_46345	*Brama japonica*	Adult
MCZ_Lot_46356	*Brama japonica*	Adult
MCZ_Lot_46357	*Brama japonica*	Adult
MCZ_Lot_46358	*Brama japonica*	Adult
MCZ_Lot_148066_1	*Brama orcini*	Adult
MCZ_Lot_148066_2	*Brama orcini*	Adult
MCZ_Lot_148381	*Caristius fasciatus*	Juvenile
MCZ_Lot_164024	*Caristius fasciatus*	Adult
USNMFIN27271	*Eumgeistus illustris*	Adult
AUMNH - caught by W. Bolliger, New South Wales 2004	*Pteraclis aesticola*	Adult
AUMNH_34777_001	*Pteraclis aesticola*	Adult
MCZ_Lot_55338	*Pteraclis carolinus*	Juvenile
MCZ_148116_1	*Pterycombus brama*	Juvenile
MCZ_Lot_76100	*Pterycombus brama*	Juvenile
MCZ_Lot_76115	*Pterycombus brama*	Juvenile
MCZ_Lot_76104_3	*Pterycombus brama*	Juvenile
MCZ_Lot_76104_4	*Pterycombus brama*	Juvenile
MCZ_Lot_76104_6	*Pterycombus brama*	Juvenile
MCZ_Lot_76111_2	*Pterycombus brama*	Juvenile
MCZ_Lot_76111_3	*Pterycombus brama*	Juvenile
MCZ_Lot_76111_4	*Pterycombus brama*	Juvenile
MCZ_Lot_76112	*Pterycombus brama*	Juvenile
MCZ_Lot_174129	*Pterycombus brama*	Adult
Fresh, custody of MC Gilbert & SH Huskey	*Pterycombus petersii*	SubAdult
MCZ_Lot_59503	*Pterycombus petersii*	Adult
MCZ_Lot_148056	*Taractes asper*	Juvenile

MCZ_Lot_161671	*Taractes asper*	Juvenile
MCZ_Lot_46347	*Taractes asper*	Adult
MCZ_Lot_46348	*Taractes asper*	Adult
MCZ_Lot_46349	*Taractes asper*	Adult
MCZ_Lot _148060	*Taractes rubescens*	Juvenile
MCZ_Lot_39974	*Taractes rubescens*	Adult
MCZ_Lot_44140	*Taractes rubescens*	Adult
MCZ_Lot_44145	*Taractes rubescens*	Adult
MCZ_Lot_46322	*Taractes rubescens*	Adult
MCZ_Lot_46325	*Taractes rubescens*	Adult
MCZ_Lot_46326	*Taractes rubescens*	Adult
MCZ_Lot_46328	*Taractes rubescens*	Adult
MCZ_Lot_46355	*Taractes rubescens*	Adult
MCZ_Lot_57531	*Taractes rubescens*	Adult
MCZ_Lot_46330	*Taractichthys longipinnis*	Adult
MCZ_Lot_46331	*Taractichthys longipinnis*	Adult
MCZ_Lot_96428	*Taractichthys longipinnis*	Adult
MCZ_Lot_131846	*Taractichthys longipinnis*	Adult
MCZ_ Uncatalogued_sp19-3	*Taractichthys longipinnis*	Adult
MCZ_Lot_150538_1	*Taractichthys steindachneri*	Juvenile
MCZ_Lot_150538_2	*Taractichthys steindachneri*	Juvenile
MCZ_Lot_44139	*Taractichthys steindachneri*	Adult
MCZ_Lot_44141	*Taractichthys steindachneri*	Adult
MCZ_Lot_44147	*Taractichthys steindachneri*	Adult
MCZ_Lot_46324	*Taractichthys steindachneri*	Adult
MCZ_Lot_46329	*Taractichthys steindachneri*	Adult
MCZ_Lot_46346	*Taractichthys steindachneri*	Adult
MCZ_Lot_46350	*Taractichthys steindachneri*	Adult
MCZ_Lot_46351	*Taractichthys steindachneri*	Adult
MCZ_Lot_46352	*Taractichthys steindachneri*	Adult
MCZ_Lot_46353	*Taractichthys steindachneri*	Adult
MCZ_Lot_46354	*Taractichthys steindachneri*	Adult
MCZ_Lot_57530	*Taractichthys steindachneri*	Adult
MCZ_Lot_57532	*Taractichthys steindachneri*	Adult
MCZ_Lot_57533	*Taractichthys steindachneri*	Adult
MCZ_Lot_57534	*Taractichthys steindachneri*	Adult
MCZ_Lot_57535	*Taractichthys steindachneri*	Adult
MCZ_Lot_172796	*Taractichthys steindachneri*	Adult

Supplemental Table 3. Summary statistics output from the BiSSE analysis. Trait state 0 indicates absence of elongated fins, state 1 indicates presence. Speciation rate ($\lambda 0$) | Speciation rate ($\lambda 1$) | Extinction rate ($\mu 0$) | Transition rate (q) | Posterior probability (p).

Parameter	lambda0	lambda1	mu0	q01	p
**Min.**	0.01203	0.0001799	3.00E-08	2.67E-05	-62.57
**1stQu.**	0.03861	0.0260566	4.64E-03	3.62E-03	-50.37
**Median**	0.04762	0.0394374	1.11E-02	6.12E-03	-49.15
**Mean**	0.04963	0.0438941	1.53E-02	7.39E-03	-49.51
**3rdQu.**	0.05858	0.05777	2.14E-02	9.89E-03	-48.25
**Max.**	0.12866	0.1810267	1.10E-01	4.36E-02	-46.84

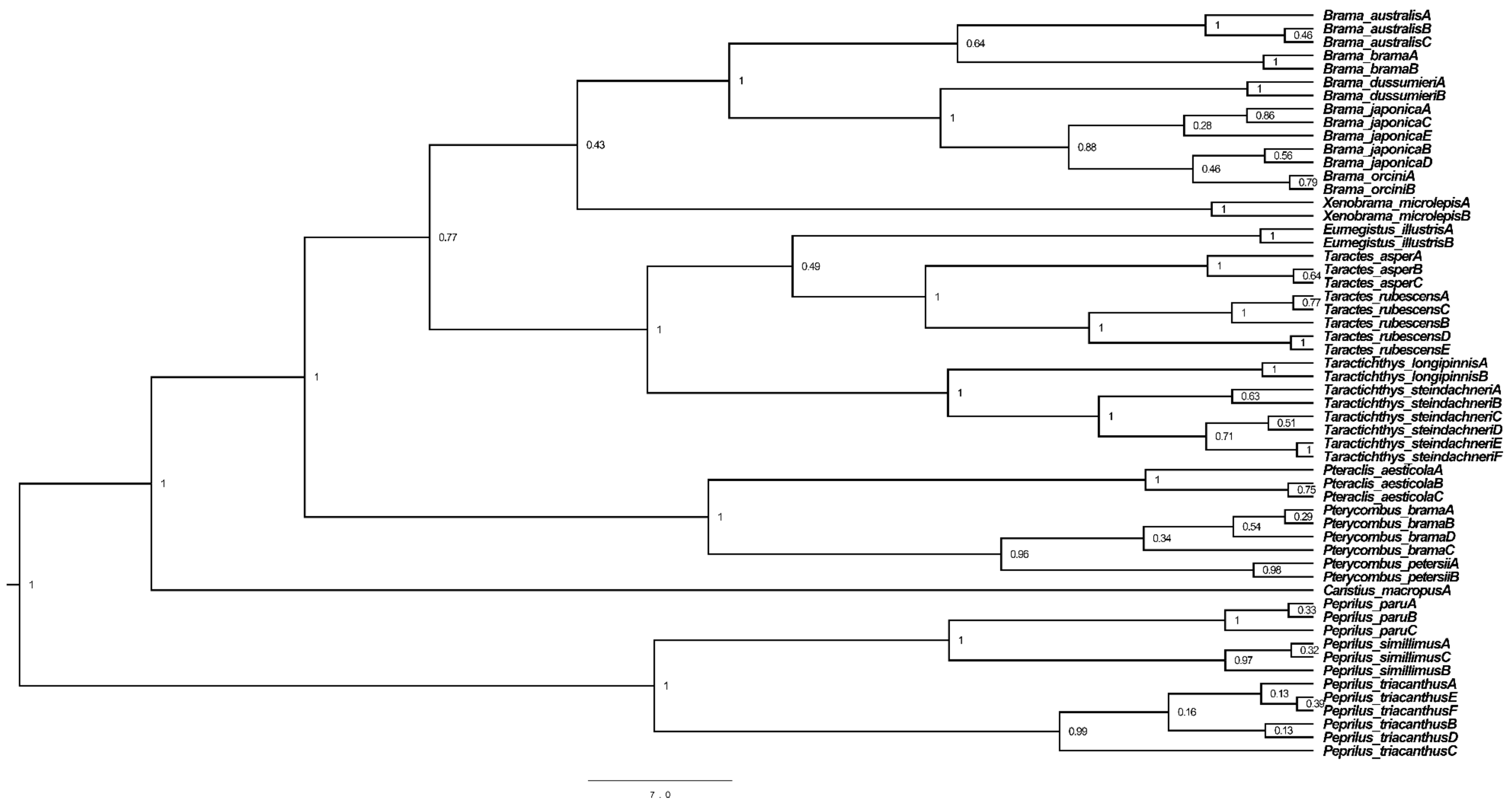

Supplemental Figure 1. Consensus of the most parsimonious topologies of the combined 640bp COI and 1141 Cytb. Values for posterior support are provided at the base of each node. For additional details, see main text.

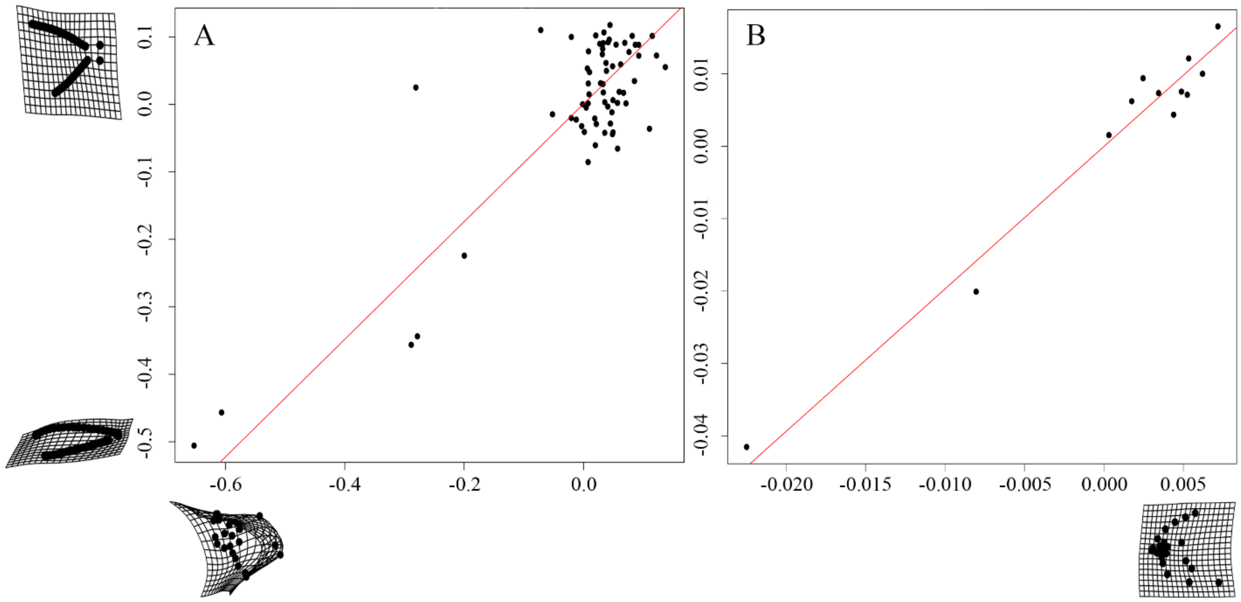

Supplemental Figure 2. Linear regression of two block partial least squares test assessing the integration of head (x-axis) and body (y-axis) shape. Covariation of head and body shape among **(A)** individuals, without accounting for the phylogeny, and **(B)** after accounting for the phylogeny.

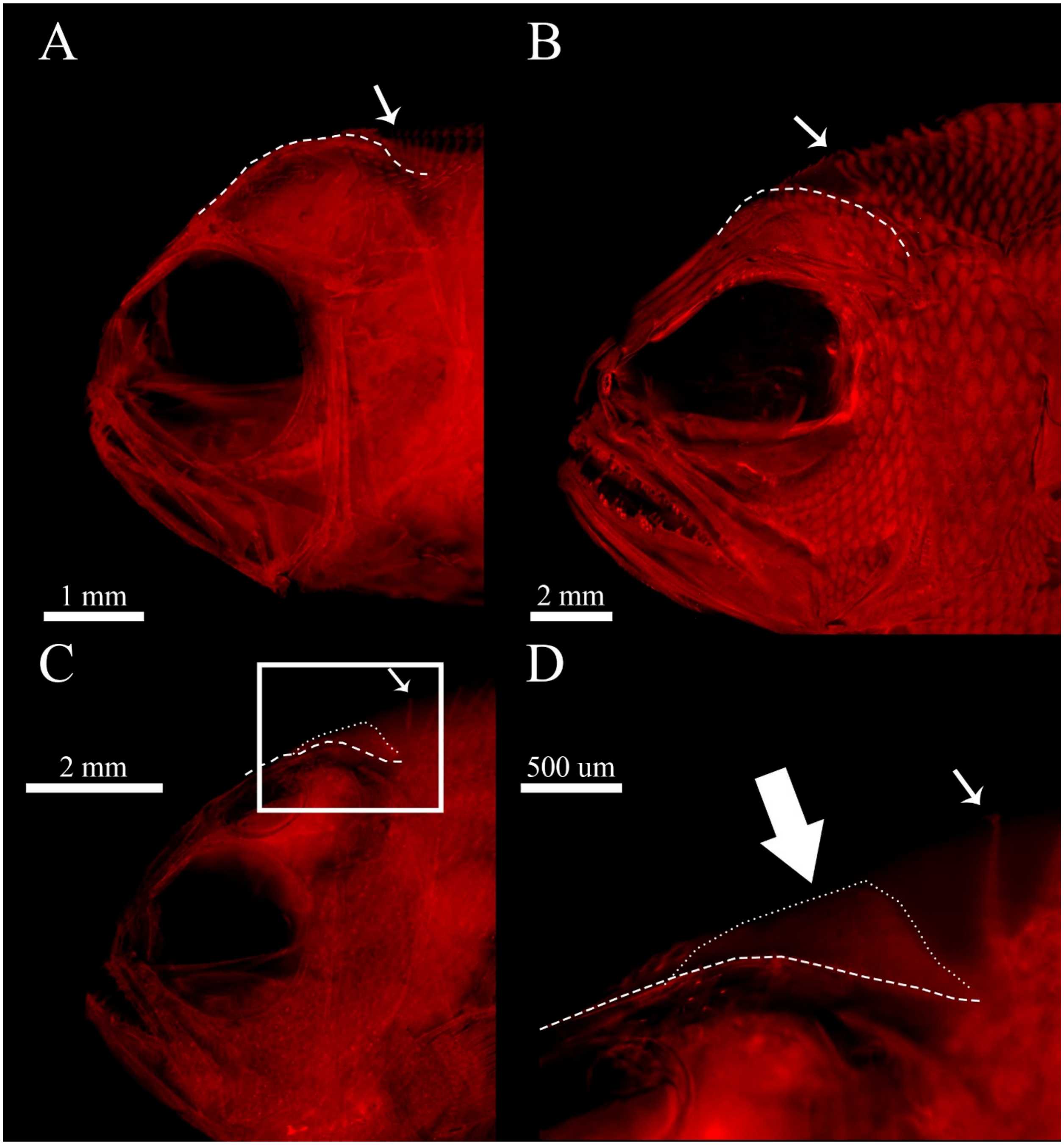

Supplemental Figure 3. Cleared and alizarin stained *Pterycombus brama* larvae **A**) juvenile **B**) and matured *Brama dussumieri* larvae (**C, D**) under fluorescent light. Dashed lines represent the dorsal profile of the neurocranium, while dotted lines outline the supraoccipital crest. *Pterycombus brama* larvae and juveniles lack any conspicuous anatomical feature indicating the development of a supraoccipital crest. Although not noticeable in non-cleared and stained specimens, pterygiophores (indicated by the smaller arrows) begin to form above the neurocranium at least as early as larval stages. *Brama dussumieri* larvae possess a relatively robust supraoccipital crest (**D**, indicated by the large arrow) and pterygiophores appear to be in their expected positions, well posterior to the back of the neurocranium.

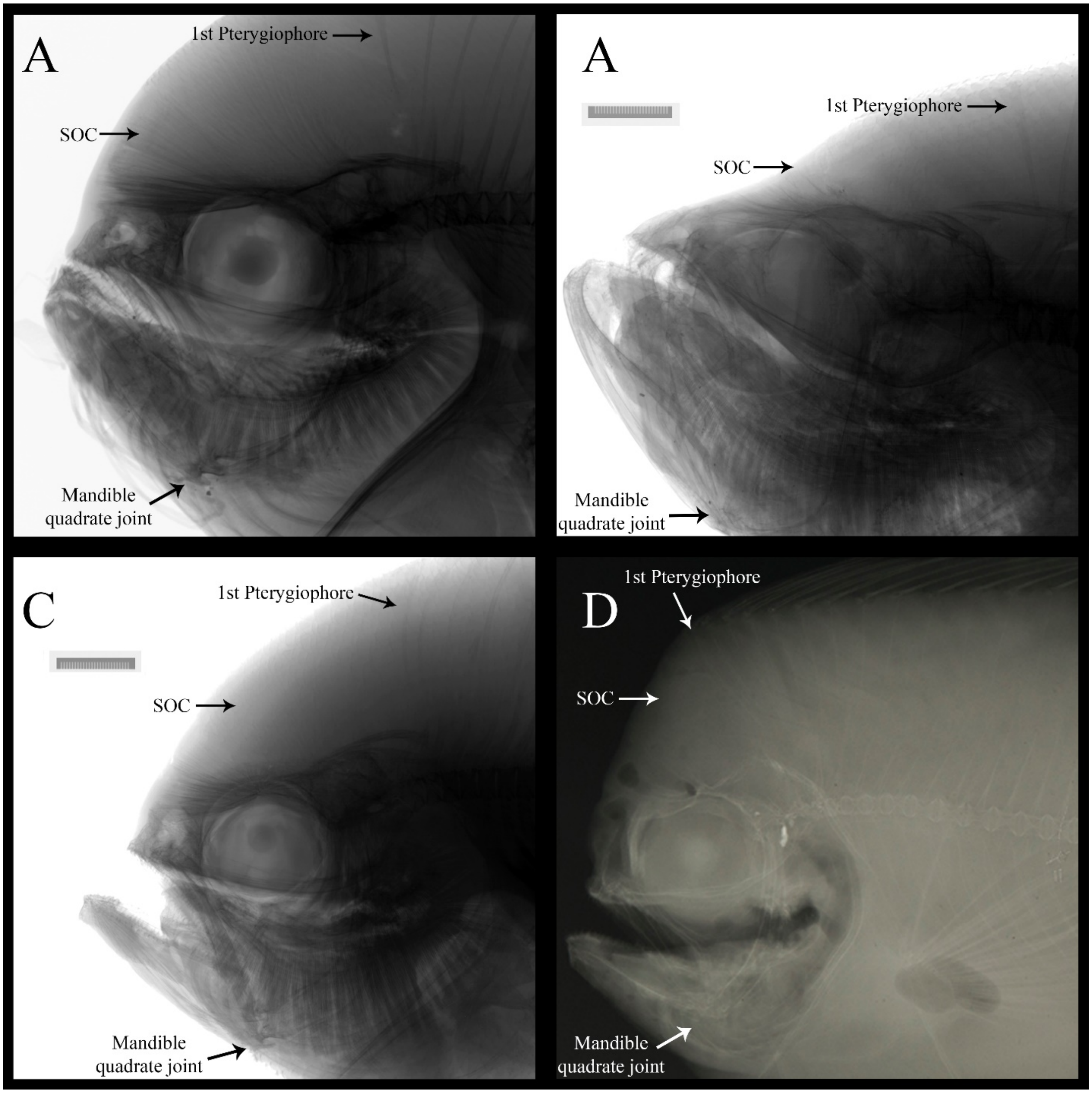

Supplemental Figure 4. Craniofacial x-rays of three bramid genera and *Caristius*. **A)** *Brama japonica* (MCZ Lot 44142), **B)** *Taractes rubescens* (MCZ Lot 46326), **C)** *Taractichthys steindachneri* (MCZ Lot 172796), and **D)** *Caristius fasciatus* (MCZ Lot 164024). X-ray images courtesy of Harvard Museum of Comparative Zoology. Image panels A-C provided by Meaghan H. Sorce, image panel D taken by Andrew D. Williston. Museum of Comparative Zoology. X-ray photography is ©President and Fellows of Harvard College.

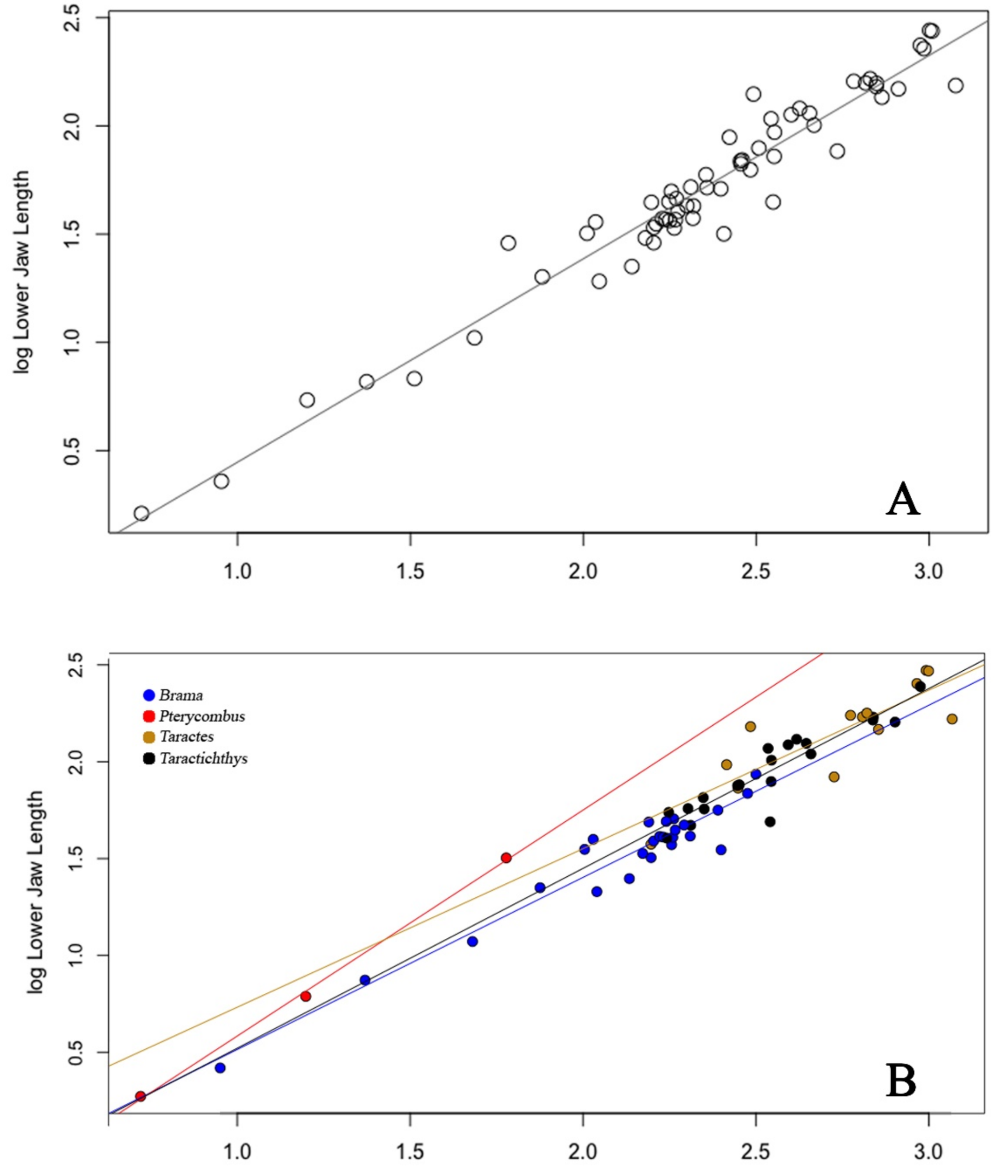

Supplemental Figure 5. Linear regression of jaw length x head length across all adult bramid specimes with an n ≥3. **A**) Linear regression of all bramid taxa together. **B**) Linear regression of each bramid genus in comparison to the other three.

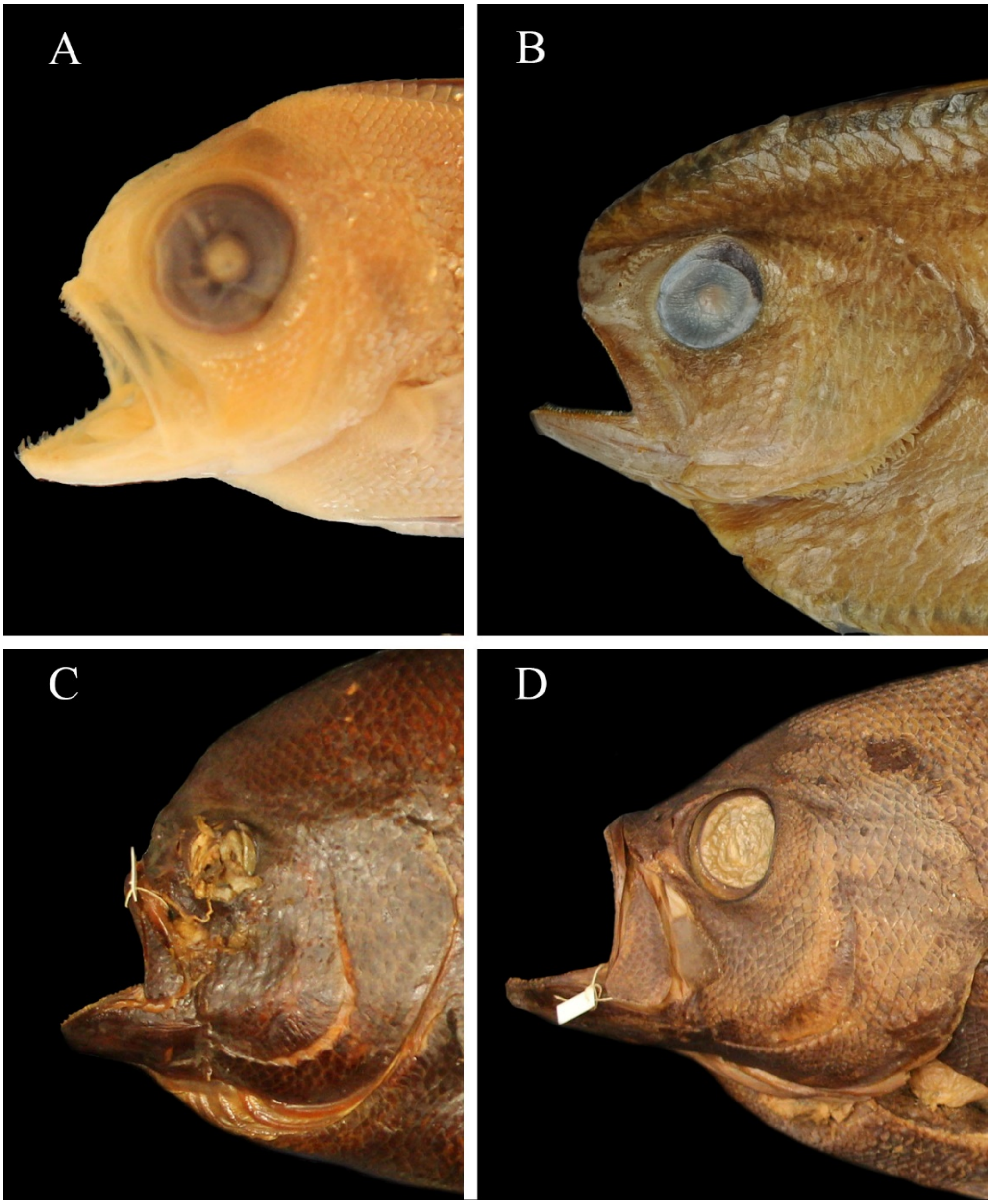

Supplemental Figure 6. Images of four fixed bramid specimens with mouths open in a fixed state. **A)** *Pterycombus brama* (MCZ Lot 76114), **B)** *Pteraclis velifera* (AUMNH I.21126-002), **C)** *Brama japonica* (MCZ Lot 46346), and **D)** *Taractes rubescens* (MCZ Lot 46325). Image panels A,C,D taken by MCG and CSL, image panel B taken by Kerryn Parkinson, Australian Museum.
